# Spatial and temporal variation of heat islands in the main urban area of Zhengzhou under the two-way influence of urbanization and urban forestry

**DOI:** 10.1371/journal.pone.0272626

**Published:** 2022-08-10

**Authors:** Yarong Yang, Fan Song, Jie Ma, Zheng Wei, Lili Song, Wei Cao

**Affiliations:** 1 School of Horticulture and Landscape Architecture, Henan Institute of Science and Technology, Xinxiang, China; 2 Henan Province Engineering Research Center of Horticultural Plant Resource Utilization and Germplasm Enhancement, Xinxiang, China; 3 Zhengzhou Forest Park, Zhengzhou, China; Feroze Gandhi Degree College, INDIA

## Abstract

Urban heat islands are major factors hindering the quality of present-day urban habitats. The ongoing acceleration of the worldwide urbanization process is leading to an exacerbation of the urban heat island effect; however, urban forestry can mitigate it. For a sustainable urban development, it is particularly important to evaluate the dual effect of both factors on the urban heat island phenomenon. In this study, we focused on Zhengzhou City (China), at the center of the Central Plains Forest City Cluster. The spatial and temporal evolutions of the local urban heat island and vegetation coverage were measured from Landsat 5 and Landsat 8 remote sensing images taken between 2006–2020 and the effects of urban construction and urban forestry on the urban heat island effect were evaluated. The results showed that, in the past 15 years, the high-temperature zone in the urban area of Zhengzhou City has gradually spread from its center to surrounding areas. Within the same period, the whole urban heat island has deteriorated and gradually improved: its area increased by 138.72 km^2^ between 2006–2014 and decreased by 135.66 km^2^ between 2014–2020. Notably, the development of vegetation coverage occurred consistently with the improvement of the heat island. A quantitative analysis of the relationship between urban construction, the urban forest, and the urban heat island has shown that factors like population density (representing urban construction), urban planning, and vegetation cover (representing the urban forest) all have an impact on the urban heat island. Based on the dynamic changes of the urban heat island in the urban area of Zhengzhou City between 2006–2020, we conclude that urban forest construction strategies are beginning to bear fruit. Overall, the findings of this study provide a theoretical basis for future urban construction and urban forest construction plans; moreover, they can support landscape pattern optimization and urban heat island mitigation.

## Introduction

With the acceleration of the urbanization process, the urban population has increased dramatically worldwide and a large number of impervious surfaces (e.g., buildings and roads) have replaced the original natural open land and vegetation [1, 2]. The most significant impact of urbanization on climate is the urban heat island (UHI) effect [3]. This relationship has long been of wide interest to the research community: the impact of urbanization factors (e.g., urban built-up areas, population, and ground surface impermeability) on UHIs have all been studied. Rizwan AM et al pointed out that the large amount of heat generated by urban structures (due to energy consumption and the reflection of solar radiation) and human activities are the main causes of UHI [4]. Urban areas are characterized by a very high degree of soil enclosure and continuous built-up areas. For instance, Italy is the European country with the highest artificial land coverage: a temporal increase of built-up coverage has been identified by Morabito M et al as the main cause of urban thermal environment deterioration [5]. The thermal performance of the asphalt concrete used in urban environments strongly regulates the urban UHI effect. Mohajerani A et al have suggested that the ongoing worldwide increase of man-made materials over land and that of anthropogenic heat production exacerbate the UHI effect [6]. He, B et al. revealed that the degree of urbanization influences the synergistic effect between heat waves and urban heat islands [7]. Moreover, in another study, they found that the urban heat island effect is stronger in larger cities and that areal agglomeration weakens the surface urban heat island effect: areal expansion worsens the thermal environment of the region [8]. This effect is becoming increasingly significant and is causing a growth of energy consumption, thermal risks, air pollution, and pollution-related mortality [9], while lowering the quality of the living environment [10, 11]. In addition to environmental and ecosystem, social and health impacts, heat-induced economic impacts should be concerned for significant economic losses related to the impacts on labours, capital, goods and services and the possible influences on population migration [8]. The urban thermal environment has become a research hotspot in the fields of urban ecology, environment, and climate: there is an urgent need for approaches capable of mitigating the risks and negative consequences of the UHI effect.

Numerous studies have shown that vegetation can reduce temperature and increase humidity by shading long- and short-wave radiation and through transpiration [12–14]. As an important part of the urban ecosystem, urban forests can effectively improve the urban substrate and have a primary role in regulating temperature and in mitigating the UHI effect [15]. Due to urban land constraints, it is impossible to rely on the increase of urban forests alone to mitigate the UHI effect: besides expanding (in a limited way) the number and scale of urban forests, it will be fundamental to maximize the efficiency of urban forest vegetation to mitigate the UHI effect and improve the ecological benefits of urban forests. Cui, Y characterized the spatial distribution characteristics of urban forests in terms of vegetation coverage, patch characteristics, and spatial patterns, exploring the relationship between the spatial layout of urban forests and the UHI effect [16]. Wang, X et al conducted a comprehensive study on the thermal environmental effects of urban forests along the horizontal and vertical directions to explain the role of urban forest structure in thermal environment regulation [17]. Li, X et al studied the effect of forest patch landscape and community structure on the cooling effect of urban forest patches. The size, shape, and spatial distribution of vegetation patches were found to have significant effects on the urban thermal environment: increasing the area and density of patches can effectively reduce the surface temperature [18]. In fact, the higher the vegetation coverage, the larger will be the cooling effect [19–21]. Therefore, building urban forests has become an important approach to mitigate the UHI effect [22]. Further analyzing the relationship between urban forests and the UHI effect can lead to new ideas and solutions for solving urban development problems.

The urbanization of Zhengzhou City (capital of the Henan Province, China) is progressing rapidly. In the past 40 years, the urban built-up area in the central part of the city has increased by more than six times; as a result, the land use pattern has changed significantly. The UHI effect in the city has hence attracted the attention of many scholars [23–29]. At present, Zhengzhou has a forested area of 2483.13 km^2^, with a forest coverage of 35% and a forest accumulation of 9.3 million m^3^. The ecological benefits brought by an increase of the forested area, the “green core” of the urban cluster, cannot be underestimated. Therefore, it is worth studying whether Zhengzhou’s urban forest construction has achieved significant results over the years and whether it is effective in alleviating the UHI effect during a rapid urbanization process. Being one of China’s central cities and an important national comprehensive transportation hub, Zhengzhou has a macro-regional role (i.e., it represents a connecton point between eastern and western China, as well as between northern and southern China); moreover, it is fundamental for the internal and external linkage development and communication of the Central Plains City Cluster: the city has a meso-regional location, being at the core node of the cluster’s “Grand Cross” spatial skeleton.

Rao was the first to propose the use of satellite remote sensing technology to study the heat island effect. Since then, many scholars have conducted in-depth studies on this effect in different cities based on thermal infrared remote sensing data: satellite remote sensing technology provides data with a higher spatial resolution than conventional meteorological data [27]. The urban heat island effect in Zhengzhou City has been studied from different perspectives: some scholars have explored the impact of urbanization on urban heat islands, while others have studied the impact of vegetation change and forestry development countermeasures on urban heat islands; however, none of them have considered the two-way impact of urbanization and urban forestry on urban heat islands.

In this study, based on Landsat data collected in three periods (from 2006 to 2020), we studied the usefulness of night lighting data to determine the level urbanization, as well as that of the inverse vegetation index and of the vegetation cover to quantify the urban forest. The spatial and temporal dynamics of all these aspects were considered to clarify the effects of 15 years of urbanization and urban forest construction on urban heat islands, in order to provide a basis for future urban and urban forest construction.

Overall, the objectives of this study were to: (1) analyze the spatial and temporal evolutionary characteristics of the urban heat island effect and of the vegetation cover in Zhengzhou City; (2) quantitatively analyze the relationship between night lighting data, vegetation cover, and the urban heat island effect; (3) evaluate the bidirectional effects of urbanization and urban forestry on the urban heat island effect in Zhengzhou City during 15 years (from 2006 to 2020). The results were expected to provide a scientific basis for the optimization of landscape patterns and for the mitigation of the urban heat island effect.

## Study area and data

### Study area

Zhengzhou is the capital of the Henan Province (China), located in its central-northern part (112°42’–114°14’E, 34°16’–34°58’N). It is bordered by the Yellow River to the north, the Song Mountains to the west, and the Yellow–Huai Plain to the southeast. Zhengzhou is characterized by a warm temperate continental climate with four distinct seasons and an average annual temperature of 14.4°C. The area includes a total of 35 large and small rivers, which belong to two major water systems (i.e., the Yellow River and the Huai River) and of which 150.4 km flow through the Zhengzhou section of the Yellow River. The natural vegetation zone of Zhengzhou is occupied by a mixed coniferous and broad-leaved forest with a large number of sycamore and pine trees; however, due to unreasonable urban construction, trees have been cut down and land coverage types have been forcibly changed, leading to increasingly serious environmental problems, including persistent hazy weather. These problems explain the urgency of mitigating the UHI effect in Zhengzhou.

**Fig 1:**
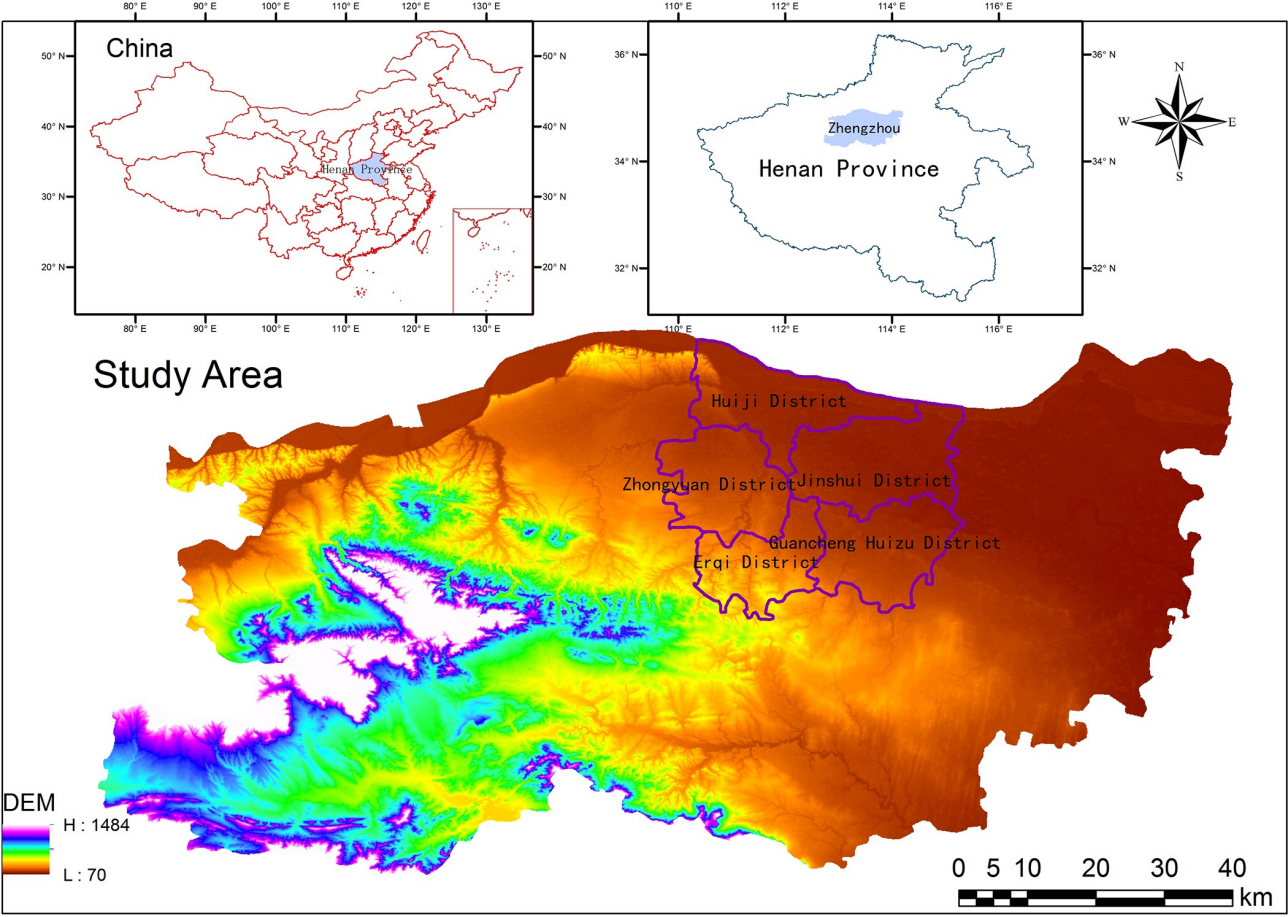
Location map of the study area.

In this study we focused on the main urban area of Zhengzhou City (Fig 1), which includes five administrative districts: the Jinshui District (located in the north-eastern part of the city), the Guancheng Huizu District (located in the south-eastern part of the city), the Huiji District (located in the northern part of the city), the Zhongyuan District (located in the western part of the city), and the Erqi District (located in the south-western part of the city).

The main urban area of Zhengzhou covers a total of 1017 km^2^; here, the population amounts to 5.225 million people and the urbanization rate is ⁓ 74.6%. In 2014, Zhengzhou City was awarded the title of China’s national forest city; moreover, in 2018, the “Forest Henan Ecological Construction Plan (2018–2027)” proposed the creation of the Central Plains Forest City Cluster by involving national and provincial forest cities. Zhengzhou, represents the “green core” of this cluster and its ecological security is guaranteed as a node city of the “One Belt and One Road” initiative. This initiative involves the construction of logistics channel hubs that connect eastern and western countries.

### Data source

The digital elevation model (DEM) data used in this study are shown in Fig 1. The satellite image strip number of Zhengzhou City in the study area was 124, the row number 36, and the projected coordinate system WGS84. Landsat 5 TM, Landsat 8 OLI, and TIRS images were chosen as data sources for the study. Based on the acquisition time and the almost complete absence of clouds, three images were selected. They were on May 16, 2006 (TM), May 6, 2014 (OLI/TIRS), and May 22, 2020 (OLI/TIRS), respectively. These data were obtained from the Geospatial Data Cloud Platform of the Computer Network Information Center of the Chinese Academy of Sciences (http://www.gscloud.cn).

To study the impact of urban construction on the UHI effect, we used night-lighting remote sensing data collected by Luojia 1 on October 30, 2018. The Luojia-1 satellite was launched and supervised by Wuhan University, and its night-lighting data were provided by the Gaofen Hubei Center (http://59.175.109.173:8888/index.html).

## Methods

### Night-lighting data

The ArcGIS software was used to pre-process (including cropping, mosaic, mask extraction, and radiation calibration) the “Luo Jia No.1” night-lighting image data. The radiation calibration formula of the “Luo Jia No.1” product was as follows [30]:

$$L={\mathrm{DN}}^{3/2}\times 10^{‐10}$$ (1)

where L is the absolute radiation correction after the radiation brightness value and DN is the image grayscale value.

### The UHI effect

Regional thermal field studies are usually based on two main indicators: the absolute bright temperature and the relative bright temperature (TR).

Here, we used a single-window algorithm to calculate the absolute bright surface temperature and used the remote sensing radiation value of a satellite’s thermal infrared channel to determine the temperature inversion [31, 32]; then, we produced a thematic map of the absolute bright temperature distribution in Zhengzhou City and related statistics. Zhengzhou City’s historical meteorological data were obtained from tianqi.com (http://www.tianqi.com/): three Landsat remote sensing images were acquired on May 16, 2006, May 6, 2014, and May 22, 2020, respectively. No precipitation occurred within three days before and after each acquisition date. The stable atmosphere and the dry air characterizing the area those days were extremely favorable to the formation of urban heat islands [24].

Concerning the thermal infrared band of TM data, the higher the brightness value of the image element, the higher will be the land surface temperature, and vice versa. The luminance temperature was calculated using TM thermal data (TM6): first, the image grayscale values (DN value) were converted into thermal radiation intensity values; then, the thermal radiation intensity values were used to obtain luminance temperature values [31]:

$$L_{\left(\lambda \right)}=0.1238+0.005632156Q_{DN}$$ (2)

where L_(λ)_ is the radiation intensity received by the TM sensor (mW cm^−2^ sr^−1^ μm^−1^) and Q_DN_ is the DN value of the image element.

$$T6=K_{2}/\ln(1+K_{1}{/L}_{\left(\lambda \right)})$$ (3)

where T6 is the image brightness temperature (K) of TM6, while K_1_ and K_2_ are constants (K_1_ = 60.776 mW cm^−2^ sr^−1^ μm^−1^ and K_2_ = 1260.56 K). In the case of Landsat 5:

$$T6=1260.56/\ln(1+60.776/(0.1238+0.005632156\times Q_{DN})$$ (4)

The radiant bright temperature of T6 was obtained under the assumption that the features were all black bodies, and that they did not reflect the real surface temperature. Previous studies have shown that the real surface temperature can be calculated from the radiance of surface features according to the following equation:

St=T6/[1+(λ×T6/ρ)/Inε]. (5)

where St represents the uncorrected ground temperature (K), λ the thermal infrared broken short central wavelength (11.5 μm; ρ = 0.014387 m K), and ε is the specific emissivity of the ground material. Since the urban subsurface is very complex, according to Griend et al. [33], ε correlates well with the normalized difference vegetation index (NDVI).


ε=1.0094+0.047Ln(NDVI). (6)

When the NDVI > 0, Eq (6) can be used to obtain ε; when NDVI < 0, the empirical value of 0.92 proposed by Weng et al. is instead assigned to ε [34].

St represents the absolute bright temperature value of the ground in a certain quadrant. This value needs to be converted to Celsius degrees befire relating it to the daily temperature experienced by the city population:

T(°C)=St−273.15 (7)

For years 2015 and 2020, we considered also Landsat 8 data, which include two thermal infrared bands (i.e., Band10 and Band11). In this case, the thermal field temperature inversion is basically the same as the that for Landsat 5 data. Since there is almost no difference in the spatial layout distribution of the regional thermal field in the inversion of the two infrared bands, but only a slight difference in the degree, we used the average of the two bands in the actual analysis.

The ground bright temperature is the temperature value for each ground image element and can be used to obtain a macroscopic spatial layout of the UHI effect; however, it does not allow a detailed quantitative regional analysis. TR represents the absolute difference of the UHI effect in the study area over time and space. Here, we applied the concept of relative heat island intensity to urban heat island classification [35, 36]. The corresponding calculation equation is as follows:

TR=(Ti−Ta)/Ta (8)

where Ti is the bright temperature at the i-th point in the study region and Ta is the average bright temperature of the study area. The criteria used for the UHI classification (based TR) are shown in Table 1 [37].

**Table 1:** Classification of the relative brightness temperature.

Heat Island Class	Green Island	Weak heat island	moderate heat island	Strong Heat Island	Extremely strong heat island
Relative bright temperature	<0	0~0.1	0.1~0.2	0.2~0.4	>0.4

### Vegetation index and vegetation coverage

NDVI and FVC are considered as two key biophysical parameters in thermal remote sensing analysis. Many parameters can be employed to characterize the condition of vegetation based on remote sensing. Among them, the normalized difference vegetation index (NDVI) can be reliably used to estimate the cooling capacity of urban forests [38], which is defined as the ratio of the difference between the near infrared and visible red band values and the sum of these two bands. Since this index is the best indicator of vegetation growth and coverage, it is often used to determine the quantitative characteristics of vegetation, seasonal changes, and land coverage. In case of Landsat 5 data, the NDVI is calculated as follows:

NDVI=(B4−B3)/(B4+B3) (9)

where B4 and B3 represent the digital number (DN) values of the TM3 and TM4 bands, respectively. The NDVI values vary within the range [−1,1]. In the case of Landsat 8 data, due to the necessity of band adjustments, the following formula should be used to calculate the NDVI:

NDVI=(B5−B4)/(B5+B4) (10)

where B5 and B4 represent the DN values of bands 5 and 4, respectively.

FVC is often used as a proxy for vegetation abundance. The vegetation coverage can be calculated based on the NDVI as follows:

$$FVC=NDVI-NDVI_{MIN}/NDVI_{MAX}-NDVI_{MIN}$$ (11)

where NDVI is the actual value of the NDVI of a certain image element; while NDVI_MAX_ and NDVI_MIN_ are the maximum and minimum values of the NDVI in the study area, respectively. Four levels of vegetation coverage were considered: (1) low coverage, fractional vegetation coverage (FVC) < 45%; (2) medium coverage, 45% ≤ FVC < 60%; (3) medium–high coverage, 60% ≤ FVC < 75%; (4) high coverage, 75% ≤ FVC < 100% [39].

### Landscape pattern index

Landscape patterns are reflect the landscape heterogeneity, but also result from the combined action of human activities and various ecological processes at different scales [40]. To analyze the evolution characteristics of the thermal landscape pattern in Zhengzhou City between 2006–2020, we selected two indicators (i.e., the total number of patches (NP) and the largest patch index (LPI)), from both patch and landscape levels [41]. The TR maps were exported to the TIFF format using the ArcGIS software; then, the landscape analysis software FRAGSTATS 4.2 was used to calculate the landscape indices for different periods.

## Results

### Absolute bright temperature changes in Zhengzhou City

**Fig 2:**
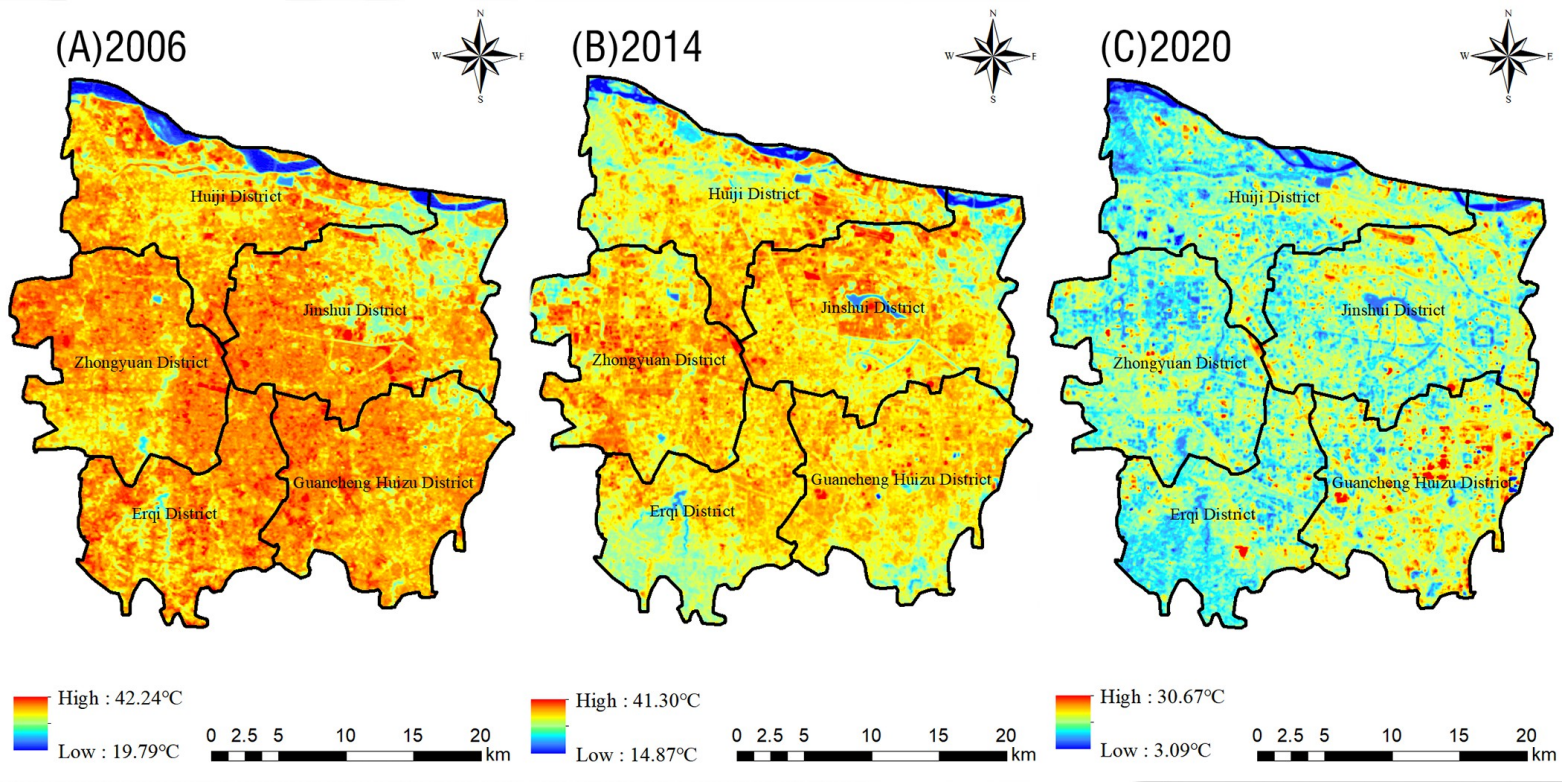
Distribution map of the absolute brightness temperature in the main urban area of Zhengzhou in 2006 (A), 2014 (B), and 2020 (C).

From the distribution of the absolute bright temperature in Zhengzhou City between 2006–2020 (Fig 2), it can be seen that the overall spatial pattern has changed greatly over the past 15 years: a high-temperature area has gradually spread from the center of the city in 2006 to the surrounding areas, and the high temperature range has expanded significantly in all administrative districts except the Erqi District (notably, the high-temperature area in the northern part of the city, near the Yellow River, has shown a change from none to something). Meanwhile, the heat increase in the central part of the city has slowed down over time. Only the Erqi District has shown a decreasing thermal trend in the past 15 years. High-temperature areas were generally concentrated as they shifted to the built-up areas: large industrial clusters and industrial parks have become the main sources of heat over the past 15 years, while lakes and wetlands have been characterized by relatively low temperatures.

**Fig 3:**
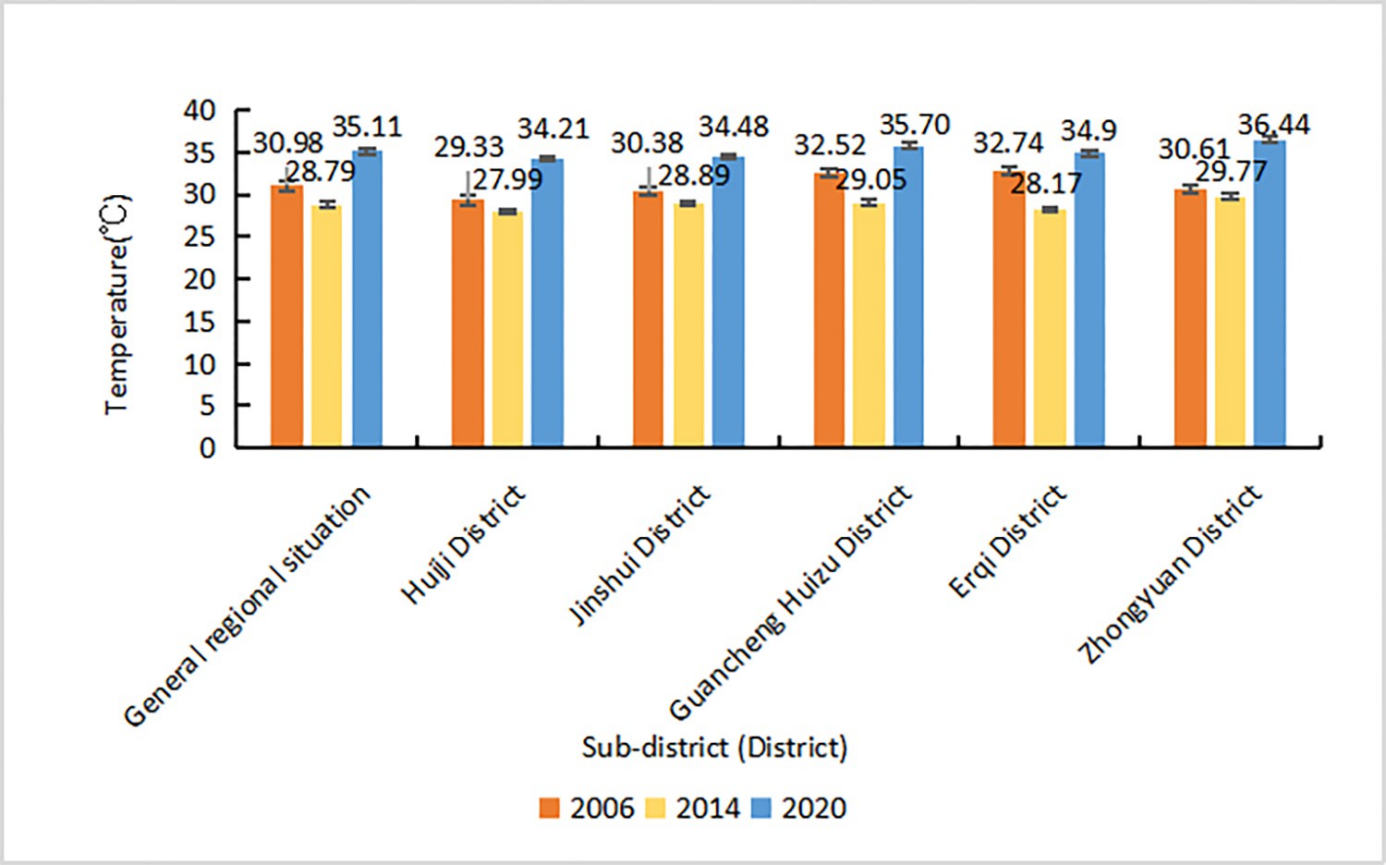
Plot of the average absolute bright temperature in Zhengzhou as a whole an in each district (between 2006–2020).

Between 2006–2020, the average surface temperature in the urban areas of Zhengzhou City first decreased and then increased, both in the study area as a whole and in the single administrative districts (Fig 3): the city’s average temperature increased by 4.65°C during this period. The average temperature increase in Huiji and Zhongyuan districts was of 4.88°C and 5.83°C, respectively: higher than that in the other administrative districts.

**Fig 4:**
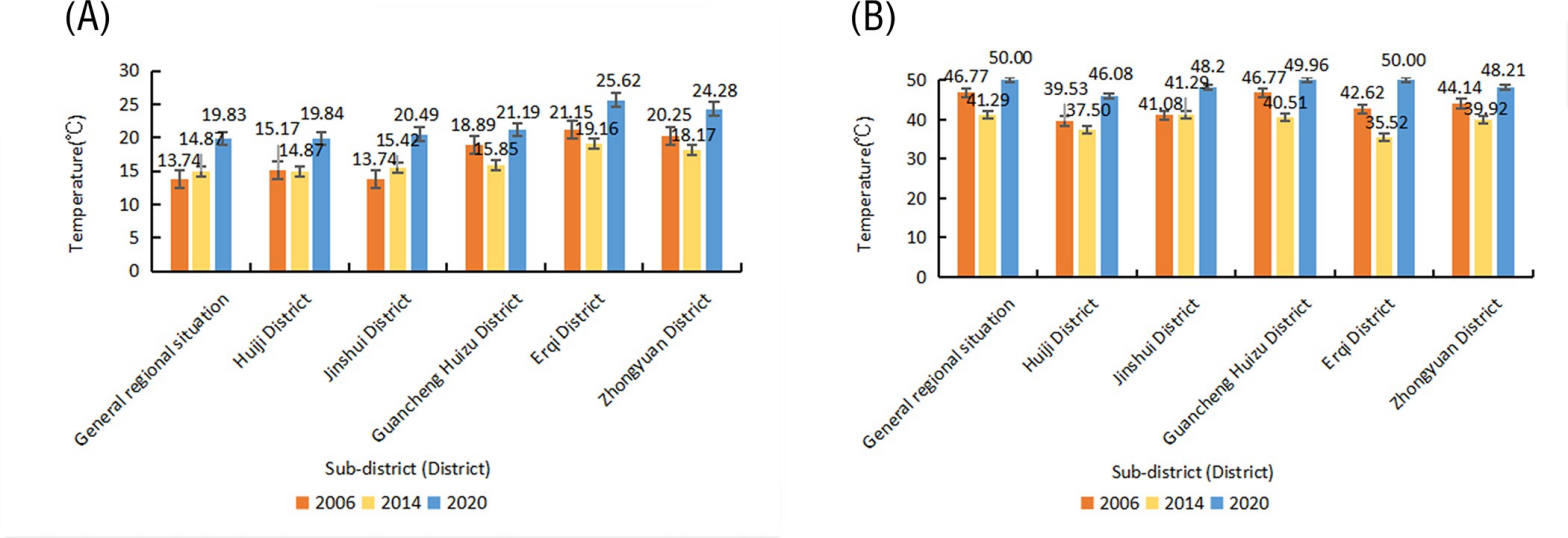
Plot of the absolute bright temperature extremes in Zhengzhou as a whole and in each district (between 2006–2020). (A) Minimum temperature, (B) maximum temperature.

In terms of temperature extremes, the overall regional minimum temperature showed a gradual increase (Fig 4A) between 2006–2020: it increased by 4.44°C. The trend observed in the Jinshui District was consistent with the overall trend for all administrative regions; however, some of these other regions showed a decline followed by an increase. The maximum temperature in the study area initially decreased over the 15-year study period (Fig 4B), but then increased: the overall increase was of 3.26°C. The highest temperatures reached in the single administrative regions were generally consistent with the overall trend. The highest increase was observed in the Erqi District, where the maximum temperature reached was 7.38°C.

### Changes in the UHI effect in the urban areas of Zhengzhou

**Fig 5:**
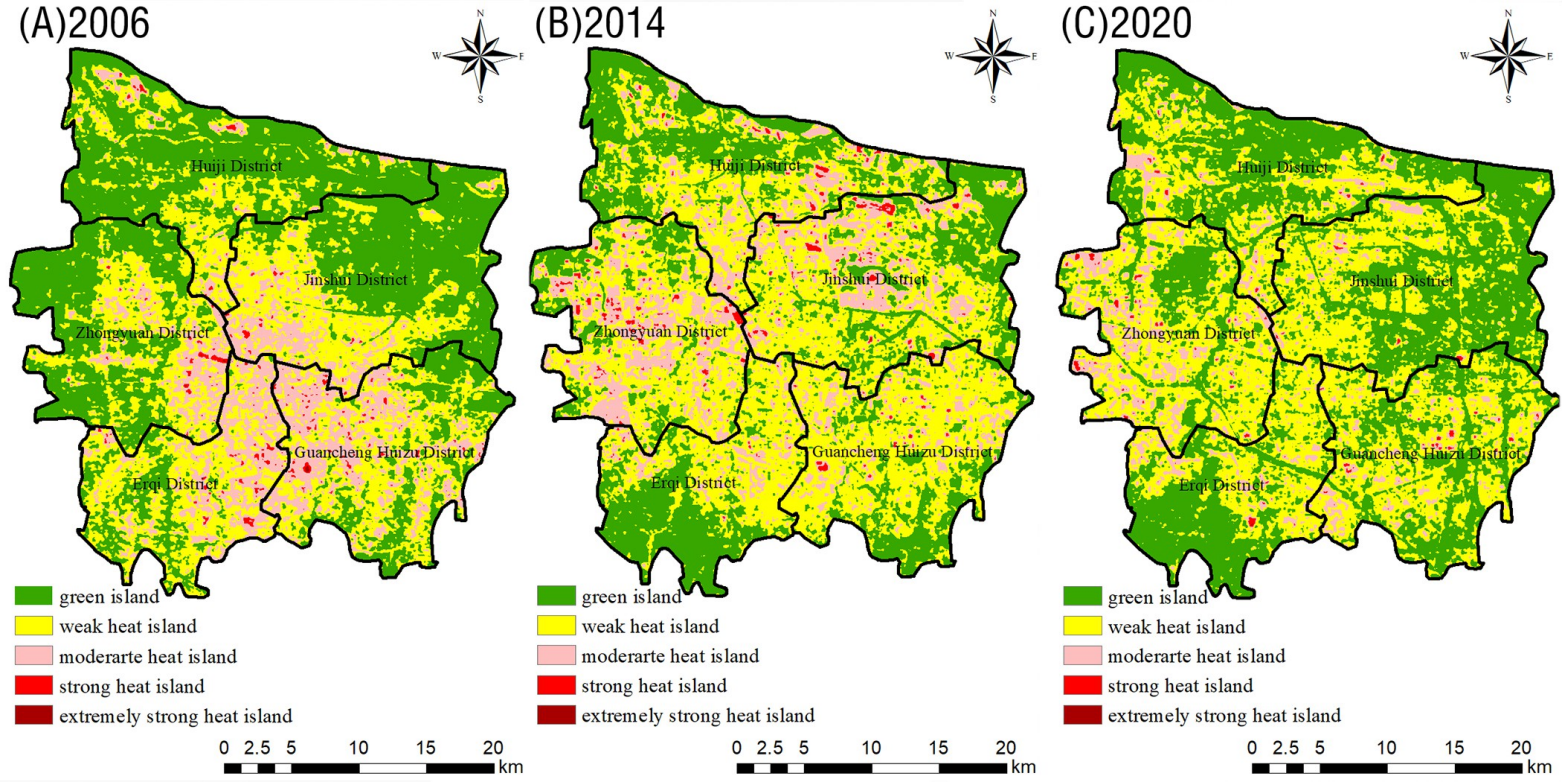
Distribution map of the relative brightness temperature in the main urban area of Zhengzhou in 2006 (A), 2014 (B), and 2020 (C).

**Fig 6:**
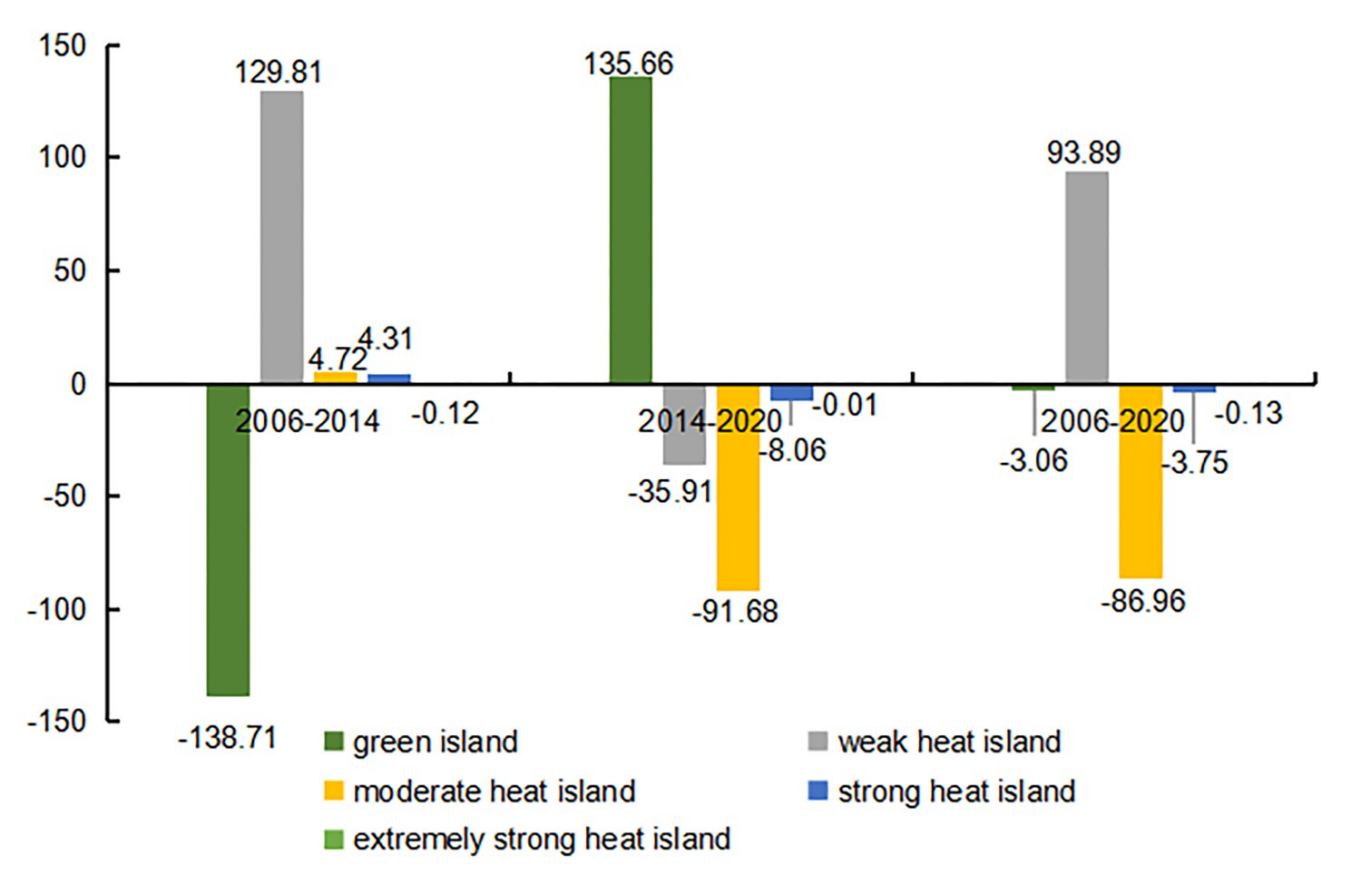
Increase and decrease of the relative brightness temperature area in the main urban area of Zhengzhou from 2006 to 2020.

The TR results indicate the green islands in the urban area of Zhengzhou gradually moved southwards from the northern part of the city during the 15-year period: their coverage increased; moreover, their distribution changed from concentrated to scattered (Fig 5). Meanwhile, weak heat islands expanded northward, moderate heat islands expanded from a continuous patch in the central part of the city to a more scattered distribution in all districts, and strong–extremely strong heat islands became more scattered. Concerning the spatial distribution of the heat field in 2020, strong–extremely strong heat islands were mainly concentrated in the central Guancheng Huizu District and in the northwestern part of the Zhongyuan District. Notably, between 2006–2020 (Fig 6), the total area of weak heat islands increased by 93.89 km^2^ (9.23%). The total area of green islands first decreased and then increased during the 15-year period; however, in 2020, it was the same as in 2006. Moderate and strong–extremely strong heat islands decreased by 85.96 km^2^, 3.74 km^2^, and 0.13 km^2^, respectively, between 2006–2020. Overall, there was a significant difference in the TR of each class of “island”: weak heat island > green island > moderate heat island > strong heat island > extremely strong heat island.

The TR data for different districts in the urban area of Zhengzhou (Table 2) show that, between 2006–2020, there was a larger proportion of green island areas in the Huiji and Jinshui districts compared to other districts. This was especially true for the Huiji District, where no extremely strong heat islands were registered. Except for the Erqi District, the areas of green islands in all regions first decreased and then increase during the 15-year period. In general, except for the Erqi District, the area of weak heat islands increased: the largest increase (of 42.41 km^2^, or 21.42%) was observed in the Zhongyuan District. The area of moderate heat islands increased trend in the Huiji and Zhongyuan districts, while it significantly decreased in the remaining regions: the largest decrease (of 43.81 km^2^, or 21.96%) was observed in the Guancheng Huizu District. The area of strong heat islands decreased in all regions except for the Zhongyuan District: the largest decrease (1.62 km^2^, or 0.81%) was observed in the Guancheng Huizu District, followed by the Erqi District (decrease of 1.61 km^2^, or 1.02%). Although the relative area increase of strong heat islands in all regions did not exceed 2%, the increase over the 15-year period is still worth of notice and should be considered a cause of concern: the areas of extremely strong heat islands in the Guancheng Huizu and Zhongyuan districts decreased over time (especially in the latter district, where extremely strong heat islands ultimately disappeared). Notably, the Erqi District was characterized by an opposite trend: the area of extremely strong heat islands increased over time.

**Table 2:** Changes in the relative brightness temperature of various regions in Zhengzhou in 2006 and 2020.

Heat Island Class	Huiji District			Jinshui District			Guancheng Huizu District			Erqi District			Zhongyuan District		
	2006	2014	2020	2006	2014	2020	2006	2014	2020	2006	2014	2020	2006	2014	2020
green island	150.35	99.334	121.565	127.087	72.704	132.622	51.609	47.788	76.930	31.993	68.119	77.947	103.646	38.021	52.562
	67.64%	44.69%	54.69%	52.47%	30.02%	54.76%	25.87%	23.96%	38.56%	20.50%	43.65%	49.95%	52.37%	19.21%	26.56%
weak heat island	60.338	90.473	85.752	84.465	119.119	101.639	87.446	129.12	107.637	79.136	73.353	67.846	68.479	97.601	110.88
	27.15%	40.70%	38.58%	34.87%	49.18%	41.97%	43.84%	64.73%	53.96%	50.71%	47.01%	43.48%	34.60%	49.31%	56.03%
moderate heat island	10.841	30.226	14.370	29.643	46.137	7.513	57.747	21.442	13.940	42.939	14.305	9.866	24.244	58.029	32.759
	4.87%	13.59%	6.46%	12.24%	19.05%	3.10%	28.95%	10.75%	6.99%	27.52%	9.17%	6.32%	12.25%	29.32%	16.55%
strong heat island	0.751	2.246	0.589	0.996	4.217	0.416	2.572	1.112	0.955	1.977	0.269	0.371	1.492	4.259	1.710
	0.34%	1.01%	0.27%	0.42%	1.74%	0.17%	1.29%	0.55%	0.48%	1.27%	0.17%	0.24%	0.75%	2.16%	0.86%
extremely strong heat island	0	0	0	0	0.02	0	0.098	0.01	0.009	0	0	0.015	0.054	0.005	0
	0%	0%	0%	0%	0.01%	0%	0.05%	0.01%	0.01%	0%	0%	0.01%	0.03%	0%	0%

### Vegetation coverage changes in the urban areas of Zhengzhou City

**Fig 7:**
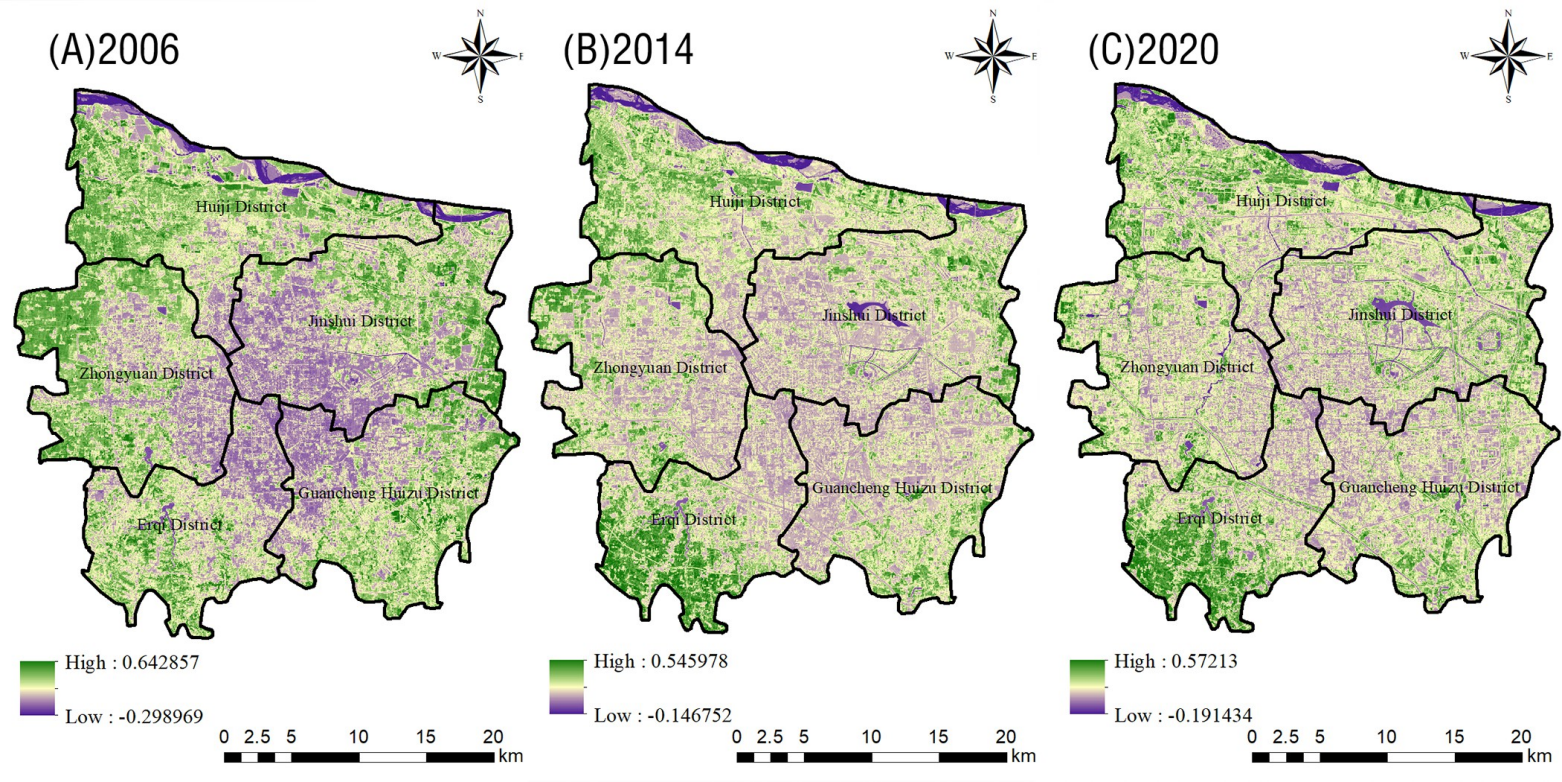
Distribution map of the NDVI in the main urban area of Zhengzhou in 2006 (A), 2014(B), and 2020 (C).

Fig 7 shows how an area of low NDVI values spread from the middle of Zhengzhou City to the surrounding areas over the 15-year period. The minimum NDVI values first increased and then decreased, and their distribution changed from concentrated to scattered, while the range increased. Meanwhile, the maximum NDVI values first decreased and then increased, and their distribution was relatively fragmented.

**Fig 8:**
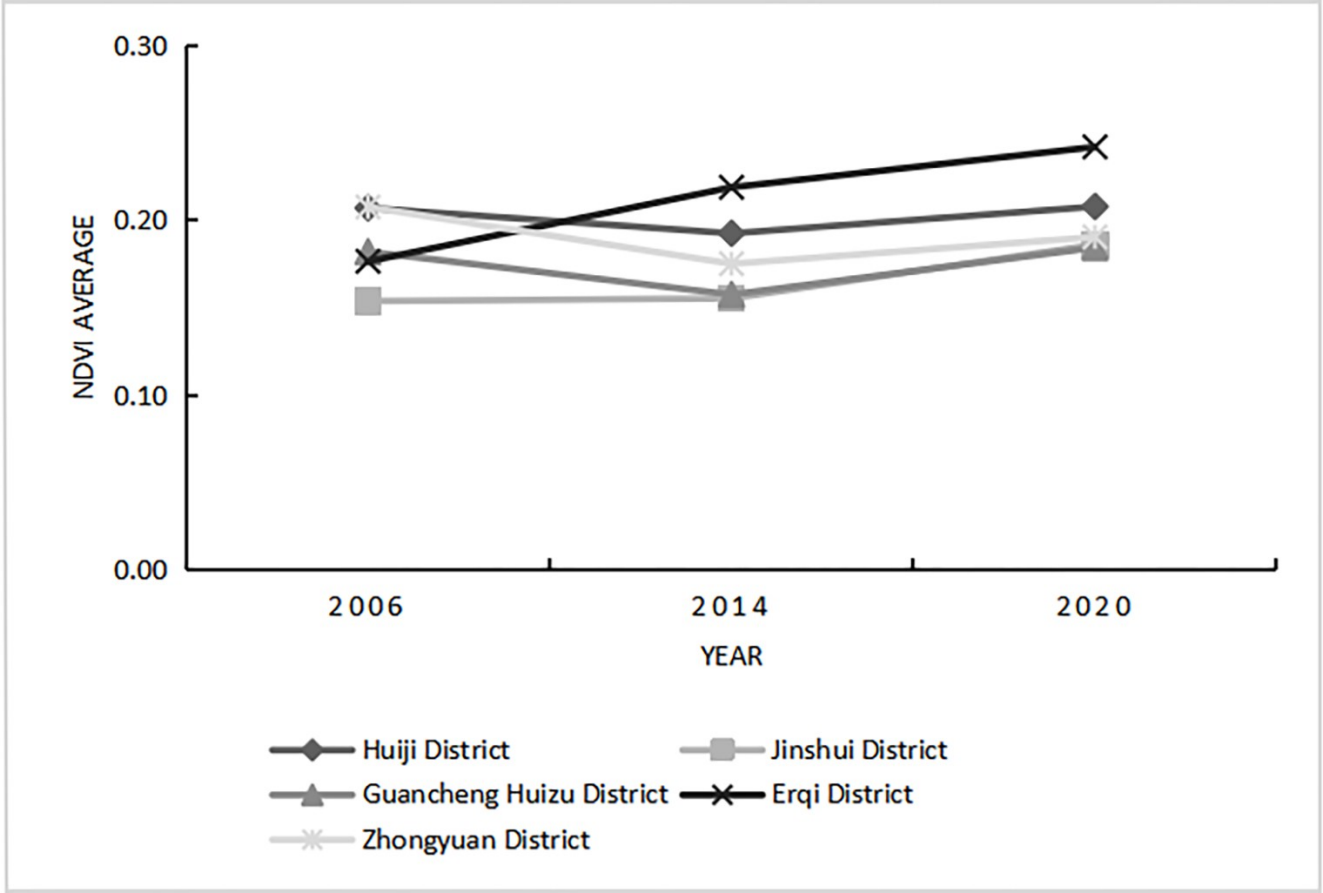
Changes in the average value of the NDVI in various urban areas of Zhengzhou from 2006 to 2020.

The mean NDVI value slightly fluctuated in all administrative districts of Zhengzhou City (Fig 8): in the Erqi District it steadily increased over the 15-year period, indicating that the ecological background and maintenance of this area were good; however, the mean NDVI values in all other districts first decreased and then increased. The average NDVI value reached its maximum in 2020 in all administrative districts, indicating that vegetation growth changed to a certain extent in the whole urban area during the 15-year period, and that urban forest construction developed well in Zhengzhou City.

**Fig 9:**
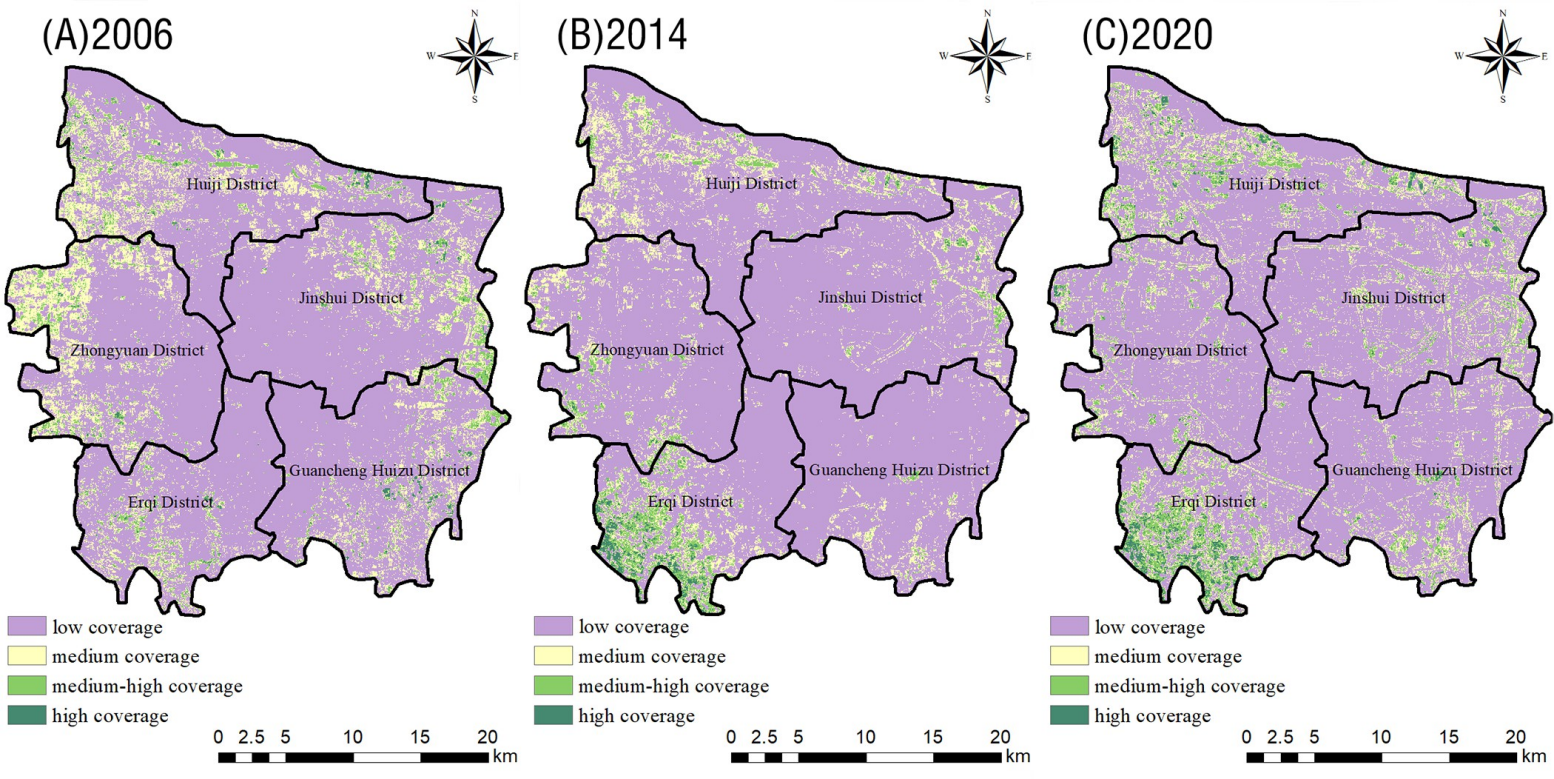
Distribution map of the vegetation coverage in the main urban area of Zhengzhou in 2006 (A), 2014 (B), and 2020 (C).

**Fig 10:**
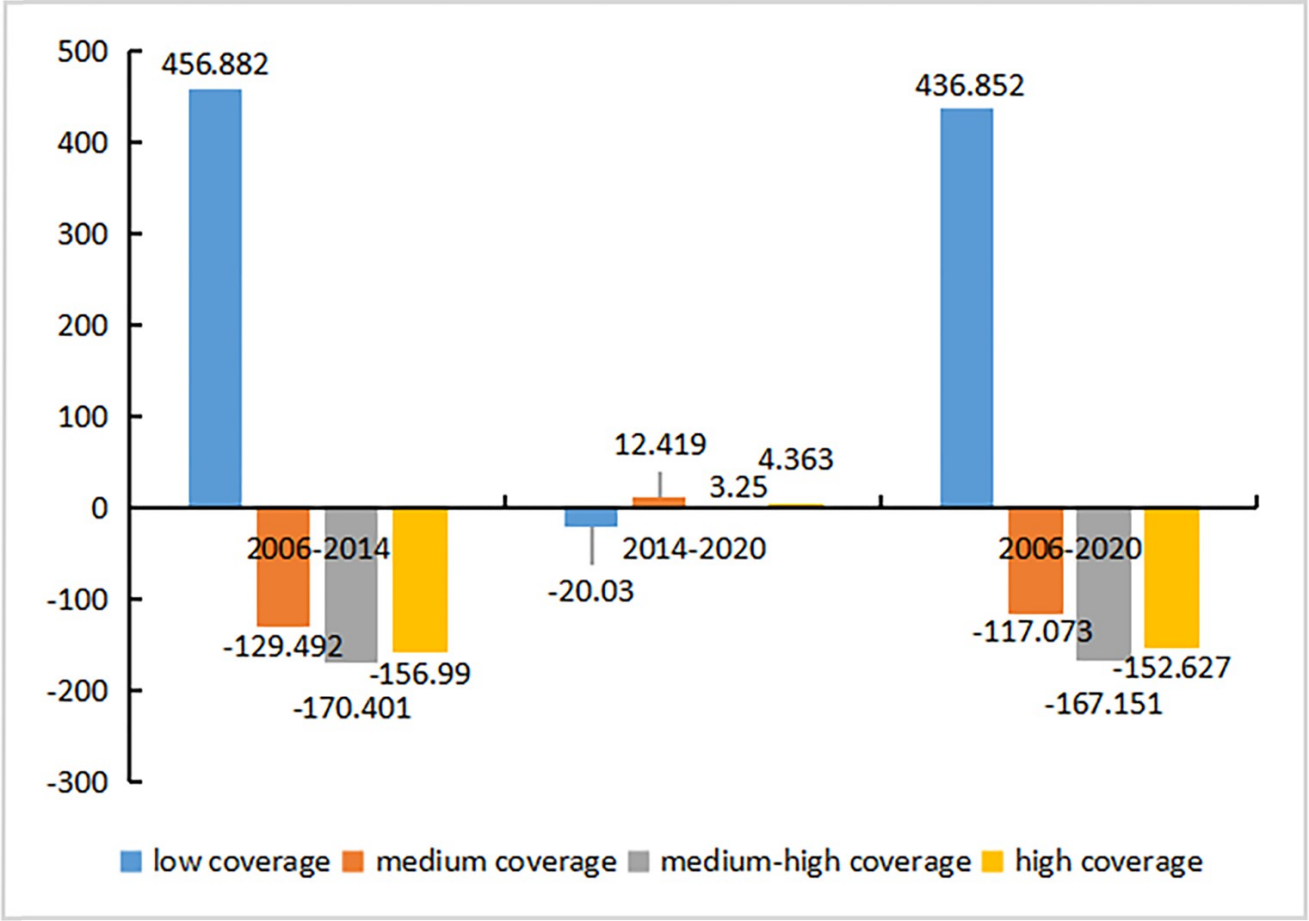
Increase and decrease of the vegetation coverage area in the main urban area of Zhengzhou City.

The vegetation coverage inversion results indicated that the low-coverage area spread from the center of the city to the surrounding areas during the 15-year period (Fig 9): it first increased significantly and then decreased, and its distribution changed from concentrated to dispersed. Between 2006–2014, there was an obvious negative change in vegetation coverage (Fig 10), highlighted by a 44.92% increase in the area of low-coverage vegetation, while the areas of all other levels of coverage decreased. However, between 2014–2020, the area of low coverage decreased by 20.03 km^2^, while the area of all other levels of coverage increased. Overall, the areal distribution of different levels of coverage reflected the following relationships: low coverage > medium coverage > medium-high coverage > high coverage.

Table 3 shows the changes in vegetation coverage that occurred in different areas of Zhengzhou City between 2006–2020. Based on these data, the vegetation coverage of the Erqi District was a clearly higher than that of other districts: here, we observed the smallest area of low coverage and the largest area of high coverage. Except for the Zhongyuan District (where the low-coverage area gradually and continuously increased), the low-coverage area generally first increased and then decreased. Among all areas of the city, the Zhongyuan District showed the largest increase (105 km^2^, or 53%). The medium-coverage area decreased instead in all regions: the largest decrease (30.75 km^2^, or 15.42%) was observed in the Guancheng Huizu District. The medium–high-coverage area also decreased in all regions: the largest decrease (47.05 km^2^, or 21.17%) was observed in the Huiji District. The area of high coverage in all regions showed decreasing trend, followed by a small increase: the largest decrease (42.34 km^2^, or 21.39%) was observed in the Zhongyuan District, followed by Huiji District (decrease of 42.7 km^2^, or 19.21%).

**Table 3:** Changes of the vegetation coverage in various regions of Zhengzhou City in 2006 and 2020.

Vegetation cover Grade	Huiji District			Jinshui District			Guancheng Huizu District			Erqi District			Zhongyuan District		
	2006	2014	2020	2006	2014	2020	2006	2014	2020	2006	2014年	2020	2006	2014	2020
low coverage	59.869	168.885	165.910	114.038	211.766	200.091	72.053	177.067	169.529	56.649	102.551	99.036	62.744	161.968	167.640
	26.93%	75.98%	74.64%	47.09%	87.43%	82.62%	36.12%	88.77%	84.99%	36.30%	65.72%	63.47%	31.70%	81.84%	84.70%
medium coverage	54.143	37.337	35.165	54.114	21.429	29.829	52.367	16.16	21.613	45.872	22.266	24.303	42.35	22.164	20.863
	24.36%	16.80%	15.82%	22.34%	8.85%	12.32%	26.25%	8.10%	10.84%	29.40%	14.27%	15.57%	21.40%	11.20%	10.54%
medium-high coverage	64.456	14.951	17.408	41.084	8.395	11.198	47.248	5.929	7.649	33.871	22.927	24.319	49.246	13.302	8.178
	29.0%	6.73%	7.83%	16.96%	3.47%	4.62%	23.69%	2.97%	3.83%	21.71%	14.69%	15.58%	24.88%	6.72%	4.14%
high coverage	43.809	1.104	3.793	32.955	0.601	1.073	27.802	0.316	0.679	19.653	8.302	8.387	43.574	0.482	1.234
	19.71%	0.49%	1.71%	13.61%	0.25%	0.44%	13.94%	0.16%	0.34%	12.59%	5.32%	5.37%	22.02%	0.24%	0.62%

### Changes in the Zhengzhou urban landscape pattern index

The landscape indices obtained from two indicators (i.e., the total NP, representing the patch level, and the LPI, representing the landscape level) for different periods between 2006–2020 (Table 4) indicate that the number of patches on green and moderate heat islands increased, while their maximum patch index decreased. Also, the number of patches on weak heat islands decreased, while their maximum patch index increased. finally, the number of patches on strong and extremely strong heat islands decreased while the maximum patch index decreased.

**Table 4:** Statistical table of the thermal landscape pattern in Zhengzhou City.

Landscape Type		NP/pc			LPI/%	
Year	2006	2014	2020	2006	2014	2020
green island	569	528	962	36.3734	28.6261	30.6646
weak heat island	788	1309	671	28.5308	0.8795	33.4756
moderate heat island	792	1043	1087	6.3806	5.0820	0.3631
strong heat island	260	337	138	0.0391	0.0402	0.0275
extremely strong heat island	6	4	2	0.0096	0.0010	0.0015

### Spatial distribution characteristics of urban night lighting data

**Fig 11:**
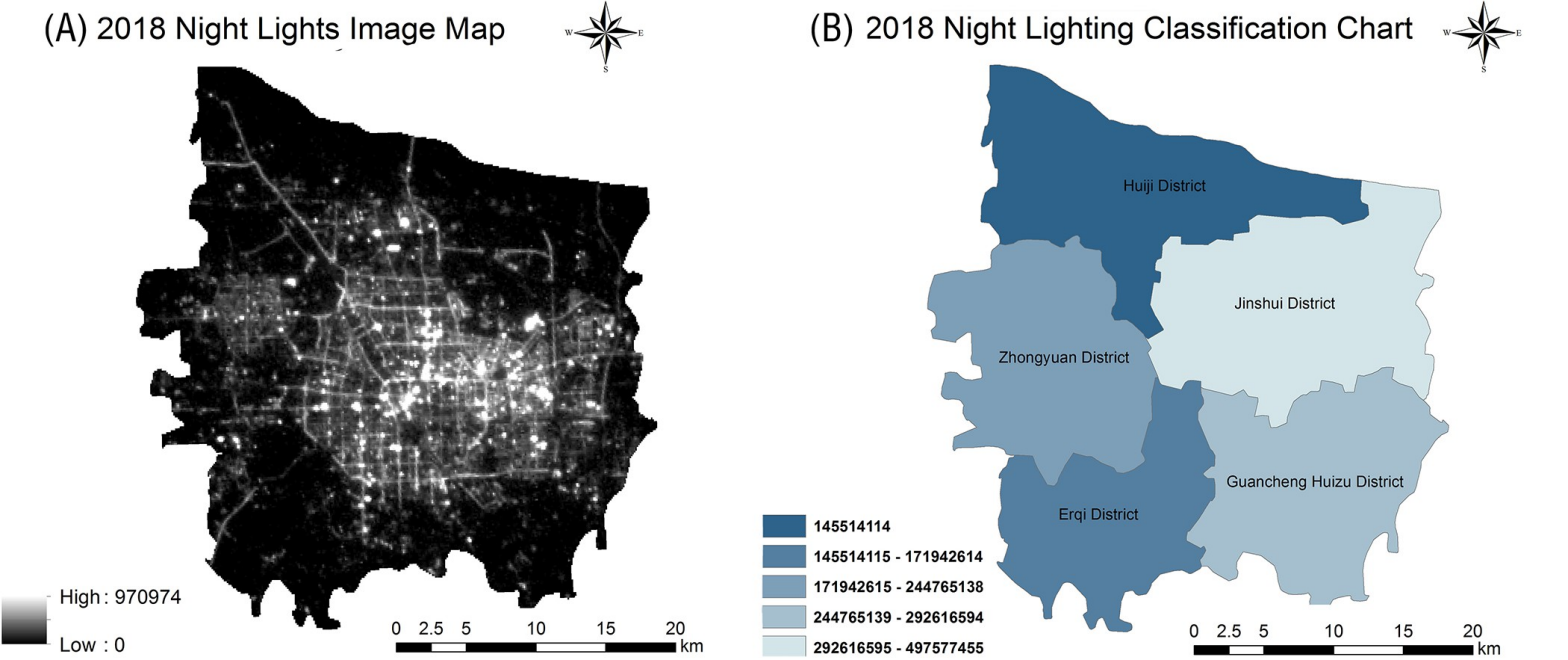
Remote sensing image and classification map of night lights in Zhengzhou city in 2018. Night light image diagram (A), night light classification diagram (B).

In the center of cities with a human population size of 500,000–1,000,000, temperature is generally 1.1–1.2°C higher than in the suburbs. The difference can even reach 1.2–1.5°C for cities with a population size >1,000,000 [42]. Intensity information, based on night lighting data, can be used to visualize the distribution of a population in real life and reflect its spatial density within a region [43]. We produced a night lighting image map of Zhengzhou City for October 30, 2018 (Fig 11A). This map shows that the central area of Zhengzhou City had a significantly higher population density than its surrounding areas. We considered the range of light and dark areas in the night lighting image map and compared the relative light temperature of Zhengzhou’s urban area between 2006–2020 in these two types of areas, finding that the intensity of the UHI effect in the light areas (presenting a larger distribution of strong–extremely strong heat islands, a high intensity of green space construction, and a higher amount of green islands) was higher than in the dark areas. After grading the night lighting data for five areas of the main urban area, we also found that the night lighting intensities of the administrative districts reflected the following relationships: Jinshui District > Guancheng Huizu District > Zhongyuan District > Erqi District > Huiji District (Fig 11B).

### Correlation analysis between FVC, the night lighting data, and the urban heat island effect in the study area

The obtained vegetation cover FVC, the night lighting images, and the relative bright temperature images of the urban heat islands in the main urban area of Zhengzhou City were normalized and 240 sample points were randomly selected from the three images; then, FVC, the night lighting data, and the relative bright temperature values of each sample were counted. The correlations among them were hence determined (results shown in Table 5): the correlation between FVC and the relevant bright temperature was extremely strong in 2006, 2014, and 2020, while the correlations between the night lighting data in 2018 and the relative bright temperature values in 2014 and 2020 were weak. This indicates that urban heat island intensity is more strictly correlated with the vegetation quality of the urban forest than to urban construction.

**Table 5:** Correlation between FVC, the night lighting data, and the urban heat island effect in the study area.

		Relative bright temperature value in 2006	2006 FVC	Relative bright temperature value in 2014	2014 FVC	Relative bright temperature value in 2020	2020 FVC	2018 Night Lighting Data
Relative bright temperature value in 2006	Pearson Correlation	1	1.000**	/	/	/	/	/
2006 FVC	Pearson Correlation	1.000**	1	/	/	/	/	/
Relative bright temperature value in 2014	Pearson Correlation	/	/	1.000**	1	/	/	0.035
2014 FVC	Pearson Correlation	/	/	1	1.000**	/	/	/
Relative bright temperature value in 2020	Pearson Correlation	/	/	/	/	1	1.000**	0.035
2020 FVC	Pearson Correlation	/	/	/	/	1.000**	1	/
2018 Night Lighting Data	Pearson Correlation	/	/	0.035	/	0.035	/	1

Note

** means that, at the 0.01 level (two-tailed), the correlation was significant.

## Analysis and discussion

The first study on Zhengzhou’s UHI is dated 1997 [44]. The authors of that study concluded that the occurrence of a UHI in Zhengzhou was mainly due to the difference between the thermodynamic properties of the urban substrate and those of the suburbs. Obvious daily, seasonal, and annual variations were observed in the intensity of the UHI effect; however, the intensity changes related to height are less apparent. Here, we compare our results with those of five previous studies: Chen, Y’s Landsat satellite remote sensing images (collected in 2000, 2004, 2008, and 2015) [25], Zhang F et al’s MODIS surface temperature data (collected between 2007–2016) [27], Min M’s Landsat images (collected in May 1996, 2000, 2006, 2010, and 2014) [26], Zhang, X’s Landsat images (collected in April–May 2000, 2008, and 2017) [28]. All of the above works concluded that the surface temperature in Zhengzhou City increased during the respective study periods and that the UHI effect became increasingly serious over time. Thich is consistent with our idea that, between 2006–2014, the overall urban thermal environment of Zhengzhou City deteriorated and the UHI effect became more serious. Since the authors of those previous studies did not consider images collected after 2017, they could not observe the reduction of the UHI effect that occurred in Zhengzhou after that time. Meanwhile, Geng, L (who selected Landsat images collected in July–August 2000, 2004, 2009, 2013, and 2019) found that, under continuous urbanization, the expansion and spreading of built-up areas, and the decrease of the green space scale, the UHI effect in Zhengzhou City did not intensify, but rather slowed down and diminished in intensity [29]. This is consistent with our idea that the urban heat environment improved between 2014–2020: urban forest construction began to bear fruit before 2020; moreover, the area of medium–high and high-vegetation coverage increased.

### Impact of urbanization construction on the UHI effect

Rapid urbanization is the main cause of the UHI effect [45–47]. We explored this relationship from two perspectives: population density and urban planning. The spatial distribution characteristics of night lights (Fig 11) suggest that: (i) a high population density may promote urban construction, including urban forestry; furthermore, population density correlates with the presence of impermeable surfaces that, in turn, correlates with the extent and intensity of heat islands; (ii) an increased emphasis on green space is key to the urbanization process; (iii) the higher the population density, the more urbanized will be a certain area, and the more funding and capacity there will be for green space development. These findings are in line with those of Xu, Y et al: a high population density would sustain an increase of the urban green space construction scale; additionally, a larger population would promote investments in green space construction by the local government [48, 49].

**Fig 12:**
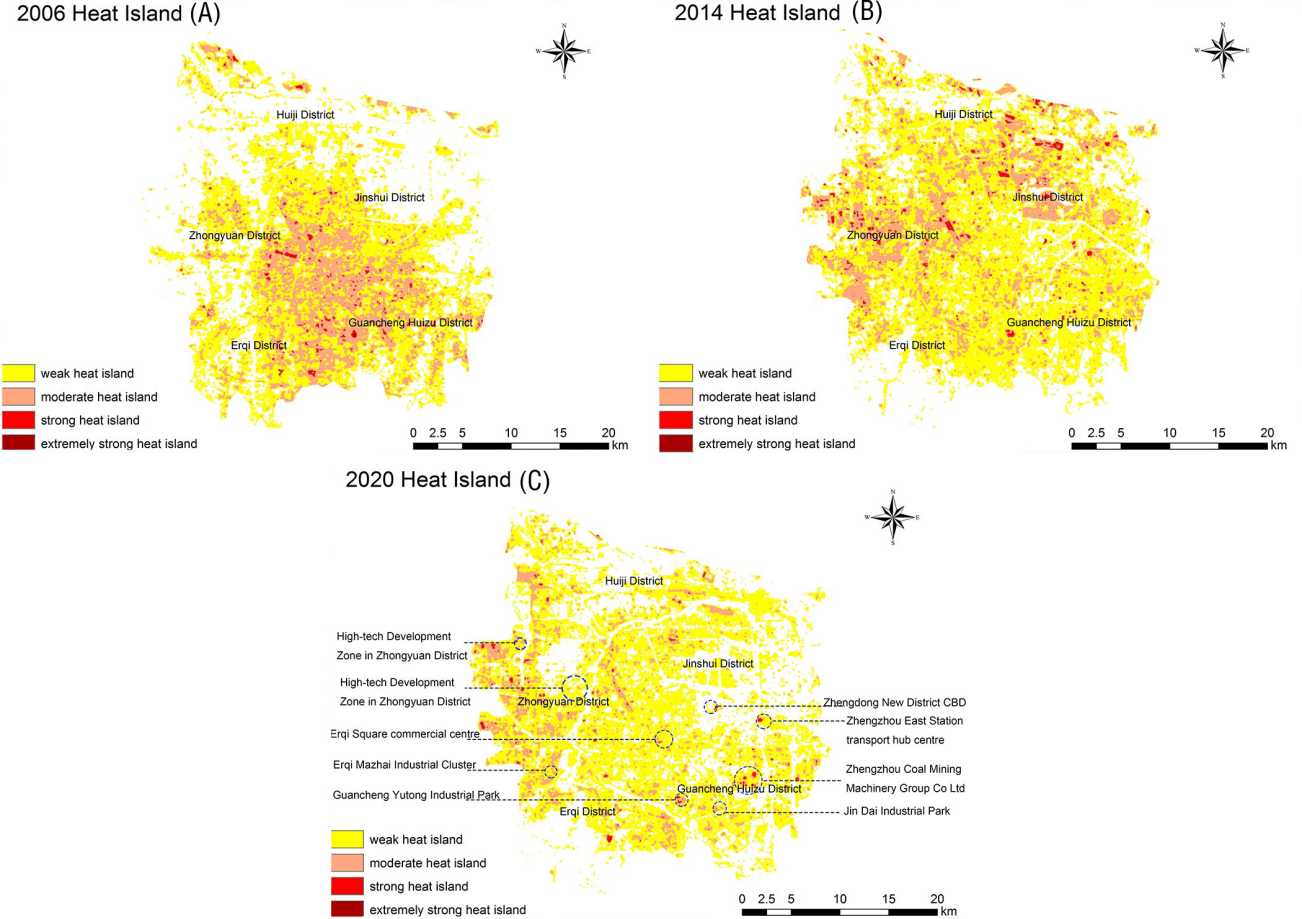
Heat island distribution in the main urban area of Zhengzhou in 2006 (A), 2014 (B), and 2020 (C).

Reasonable urban planning can effectively mitigate the UHI effect, improve the urban thermal environment, enhance human comfort, and lead to a reduction of building energy consumption [50, 51]. The Zhengzhou City Master Plan (2010–2020) (revised in 2017) proposes to build a regional-urban center system with the Erqi Square commercial center, the Zhengdong New District CBD, the Zhengzhou East Station transport hub center, and the Zhengzhou Airport transport hub center as the core, while Zhengzhou actively builds industrial clusters in the context of industrial transfer. The main areas include: the High-tech Development Zone in Zhongyuan District, Erqi Mazhai Industrial Cluster, Guancheng Yutong Industrial Park, Jin Dai Industrial Park, and Huiji District Economic Development Zone (Fig 12). An analysis of the evolution of the heat landscape pattern in Zhengzhou City over the 15-year period (2006–2020) shows that the number of weak and moderate heat island patches increased significantly between 2006–2014, while their maximum patch index decreased, indicating that the weak and moderate heat island patches gradually tended to become fragmented and complex, which is consistent with the start of large-scale construction in the city during this time period. The maximum patch index of the strong and extremely strong heat islands shows an overall decreasing trend over the 15-year period, and the number of patches is also gradually decreasing, which indicates that the urban construction of Zhengzhou City is accelerating, and the strong and extremely strong heat island patches are gradually decreasing through reasonable design and planning, increasing greenery and water area and other related measures. The layout of industrial parks is closely related to the heat island effect, and the spatial distribution of heat island patches matches the spatial distribution of industrial clusters to a high degree: the two extremely strong heat island patches are the Zhengzhou Luyuan Kitchen Waste Treatment Plant in Erqi District and the Dongfeng Nissan Zhengzhou Engine Plant in Guancheng Huizu District. The strong heat island patches are mainly located in the vicinity of the car repair center in Jinshui District, the GREE Industrial Park in Zhengzhou High-tech Zone, Zhong Yuan District, Zongye Da Intelligent Manufacturing Industrial Park, and Hengtian Heavy Industry Co Ltd, and industrial clusters and industrial parks, such as Zhengzhou Coal Mining Machinery Group Co Ltd, Henan Bonded Logistics Centre, and Economic Development Zone in Guancheng Huizu District.

### Impact of urban forest construction on UHI

Building urban forests is an important means of promoting urban ecosystem restoration and improving the human environment in China [52]. Policy guidance is important to reduce the heat island effect and influence vegetation coverage [53–55]. From the results of the study, it is clear that 2014 was an important point of change in the construction of urban forests in Zhengzhou. Previously, although NDVI was on the increase, the significant increase was only in the area of low vegetation coverage, with a decrease in the area of medium-high and high vegetation coverage, which did not provide effective relief to the UHI. after 2014, due to the increasing emphasis on ecological civilization in China, Zhengzhou was awarded the Chinese “National Forest City” for its urban forestry achievements, the construction of the Central Plains Forest City Cluster proposed in the “Forest Henan Ecological Construction Plan (2018–2027)”; the “Zhengzhou National Central City Forest Ecosystem Plan (2019–2025)” proposed that Zhengzhou will focus on creating a forest isolation circle around the city’s suburbs and vigorously promote tree planting and greening projects on the periphery of the main urban area; the “Forest Zhengzhou Ecological Construction Plan (2020–2035)”. The “Forest Zhengzhou Ecological Construction Plan (2020–2035)” promotes Zhengzhou to leapfrog from a “green city” to a “green capital”. It is clear that, between 2014–2020, the area of medium to high vegetation coverage in the urban areas of Zhengzhou increased, and the UHI effect improved and developed for the better. This shows that in the process of urban forestry, we should not only focus on the increase of the vegetation area, but also on the quality of planting and vegetation density.

**Fig 13:**
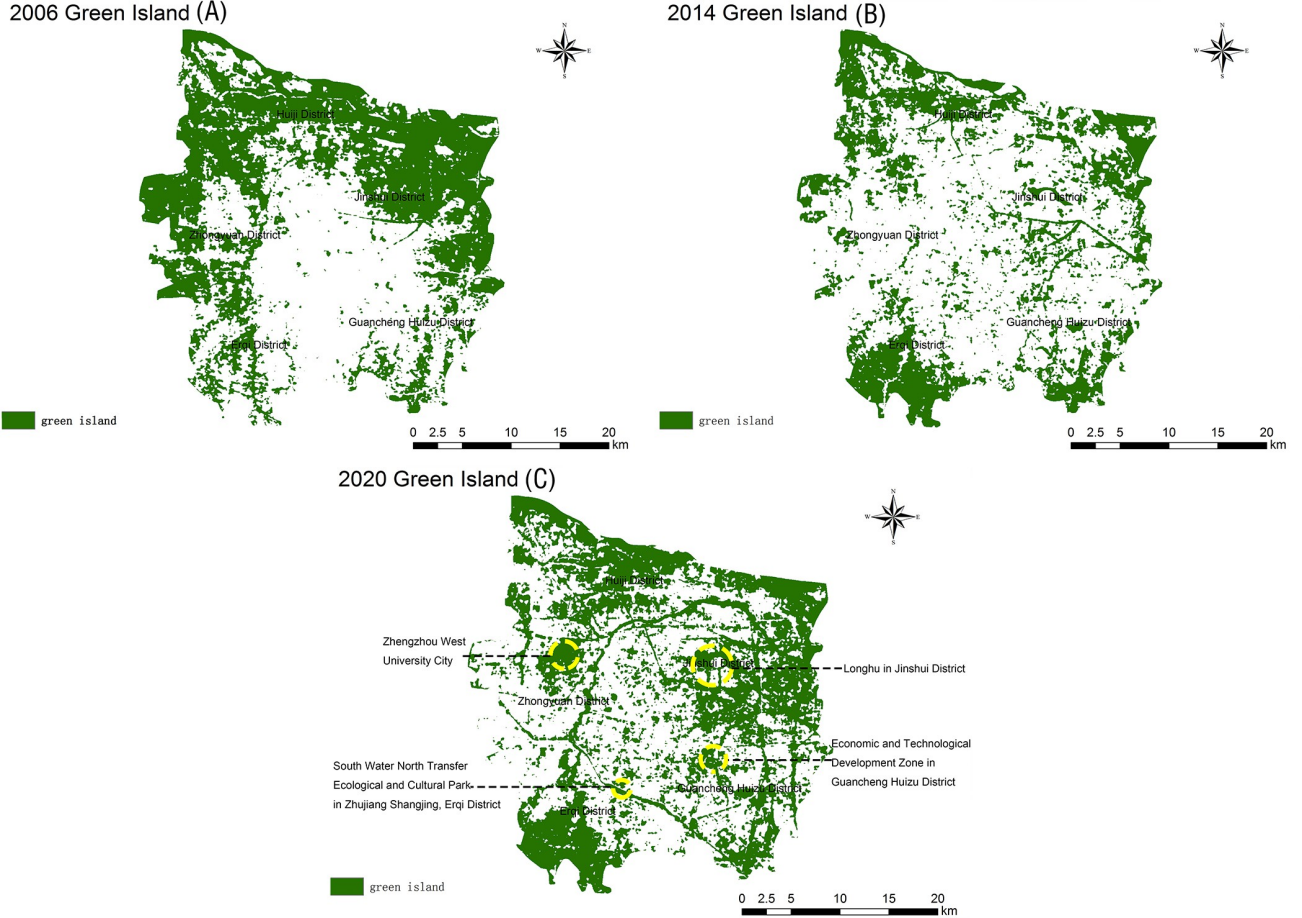
Green island distribution in the main urban area of Zhengzhou in 2006 (A), 2014 (B), 2020 (C).

The Zhengzhou City Master Plan (2010–2020) proposed the construction of new parks and green areas: large comprehensive parks and special parks, strip parks along city roads and waterways, parks and green areas in residential areas (according to the national standards), and small gardens and street green areas (according to the 500-m service radius rule). According to Table 4, the number of green islands in the urban area of Zhengzhou increased between 2006–2020 (by a total of 393 patches). By combining these data with the distribution of green islands in the urban area of Zhengzhou (Fig 5), we determined the changes in NP between 2006–2020: the number of green island patches increased over time and their distribution became more dispersed from the periphery to the center, reflecting a “greening at the seams” development. Green islands showing obvious changes were those near the Longhu in Jinshui District, the Zhengzhou West University City, the Economic and Technological Development Zone in Guancheng Huizu District, and the South Water North Transfer Ecological and Cultural Park in Zhujiang Shangjing (Erqi District) (Fig 13). The fragmentation of green islands in the main urban area of Zhengzhou has led to the creation of many small discrete patches: large dominant patches have been constantly replaced and impacted by expanding discrete urban construction sites. Studies have shown that an increase of the urban green space area can effectively mitigate the UHI effect [56, 57]. Out analyses of the spatial and temporal changes in the heat field and vegetation coverage of the urban area of Zhengzhou City showed that the vegetation condition in the study area gradually deteriorated and the intensity of the UHI effect increased between 2006–2014. In the subsequent seven-year period, the vegetation condition in the study area began to improve: the total green island area increased, while the overall intensity of the UHI effect decreased. Based on these observations, we suggest to implement a combination of greening means (according to the local conditions of Zhengzhou City) to increase the green coverage of the city and effectively mitigate the UHI effect.

### Typical point change analysis

**Fig 14:**
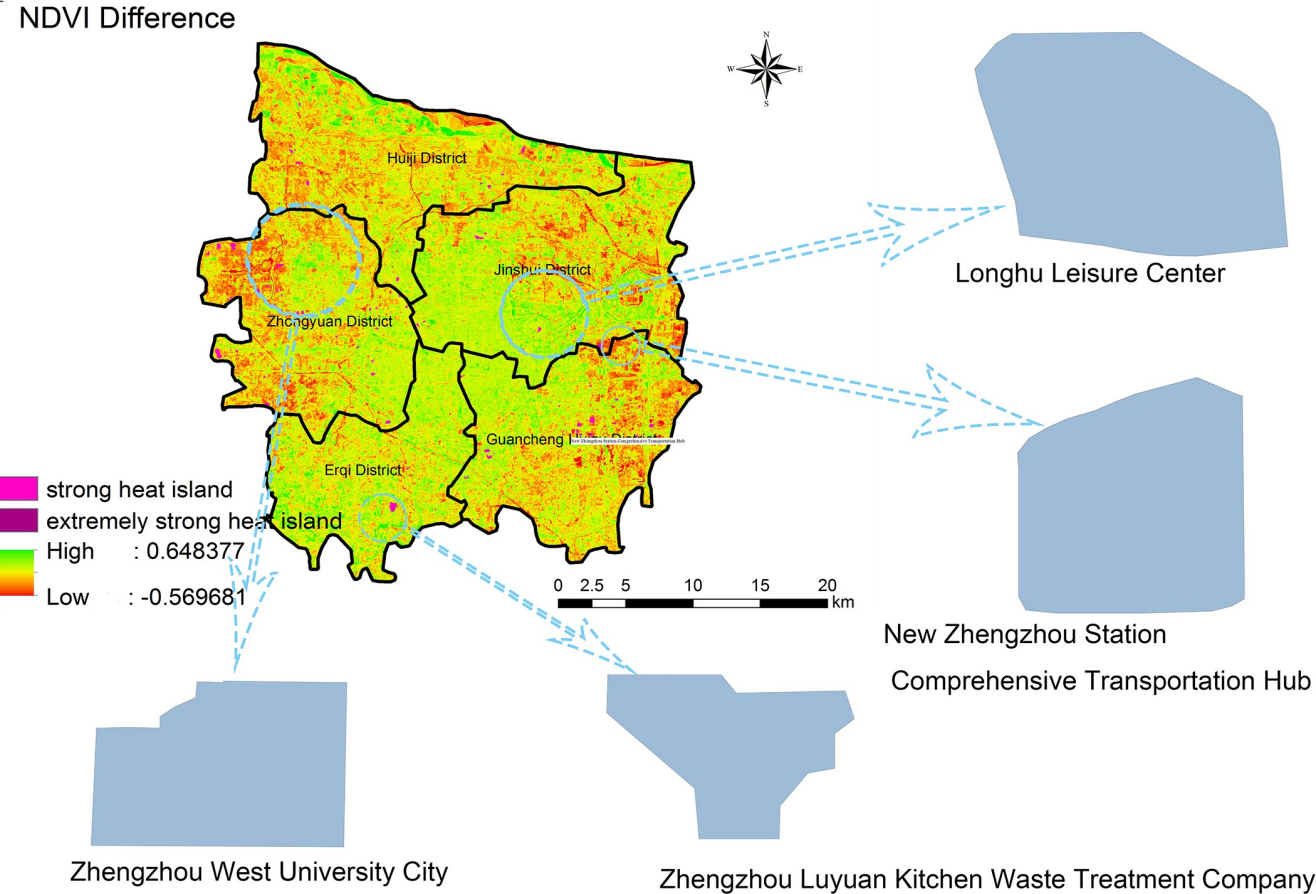
NDVI difference and typical point selection map in the main urban area of Zhengzhou in 2020 and 2006.

By considering the NDVI values obtained for 2020 and 2006 (Fig 14), we found that the area with the smallest difference was characterized by both strong-heat and extremely strong heat island patches. However, some areas with high amounts of vegetation also presented some heat island patches; this is because urban construction is typically faster than urban forest construction. According to the green island area, the strong-heat and extremely strong heat island areas identified from Figs 12 and 13, and the urban planning of recent years, we selected the Long Lake Recreation Center, the Zhengzhou West University Town, the New Zhengzhou Station Comprehensive Transportation Hub, and the Zhengzhou Luyuan Kitchen Waste Treatment Company as typical change points and further analyzed the local changes in the UHI and in the urban forest.

**Fig 15:**
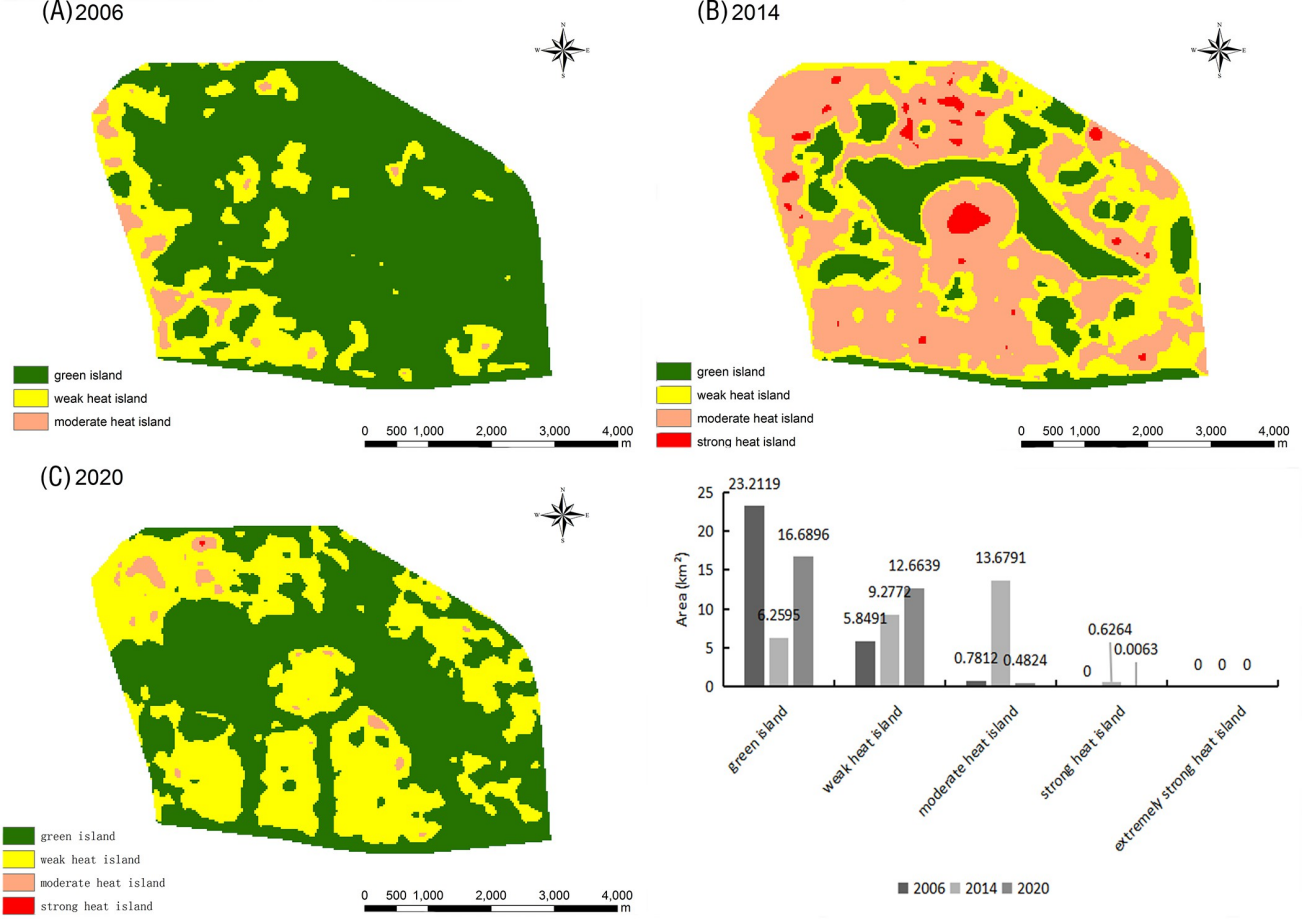
Relative brightness temperature distribution map and area change map of the Long Lake Recreation Center in 2006 (A), 2014 (B), and 2020 (C).

Located in the Jinshui District, the Long Lake Recreation Centre has a water area of ⁓ 5.6 km^2^ and was officially impounded in 2012 by the Long Lake Transfer Project. Changes in TR during the study period are shown in Fig 15. Notably, the ecology of the Long Lake Recreation Centre had not yet been destroyed in 2006. According to the Zhengzhou Urban Master Plan (2010–2020), the Long Lake area transformed into a key construction area in Zhengzhou and landscape design started along the Long Lake in 2013. Hence, the green island area around the Long Lake decreased from 2014 onwards, moderate heat island areas increased and some strong heat islands appeared. By 2020, the ecological environment of the Long Lake’s recreational center had recovered, the green island area had rebounded significantly, and the area of strong heat islands had significantly reduced. This indicates that, even though the Long Lake area is still under construction and development, an urban forest has vigorously developed: vegetation and water bodies have mitigated the heat island effect. Therefore, the ecological environment of the Long Lake has not been greatly affected and extremely strong heat islands have never appeared.

**Fig 16:**
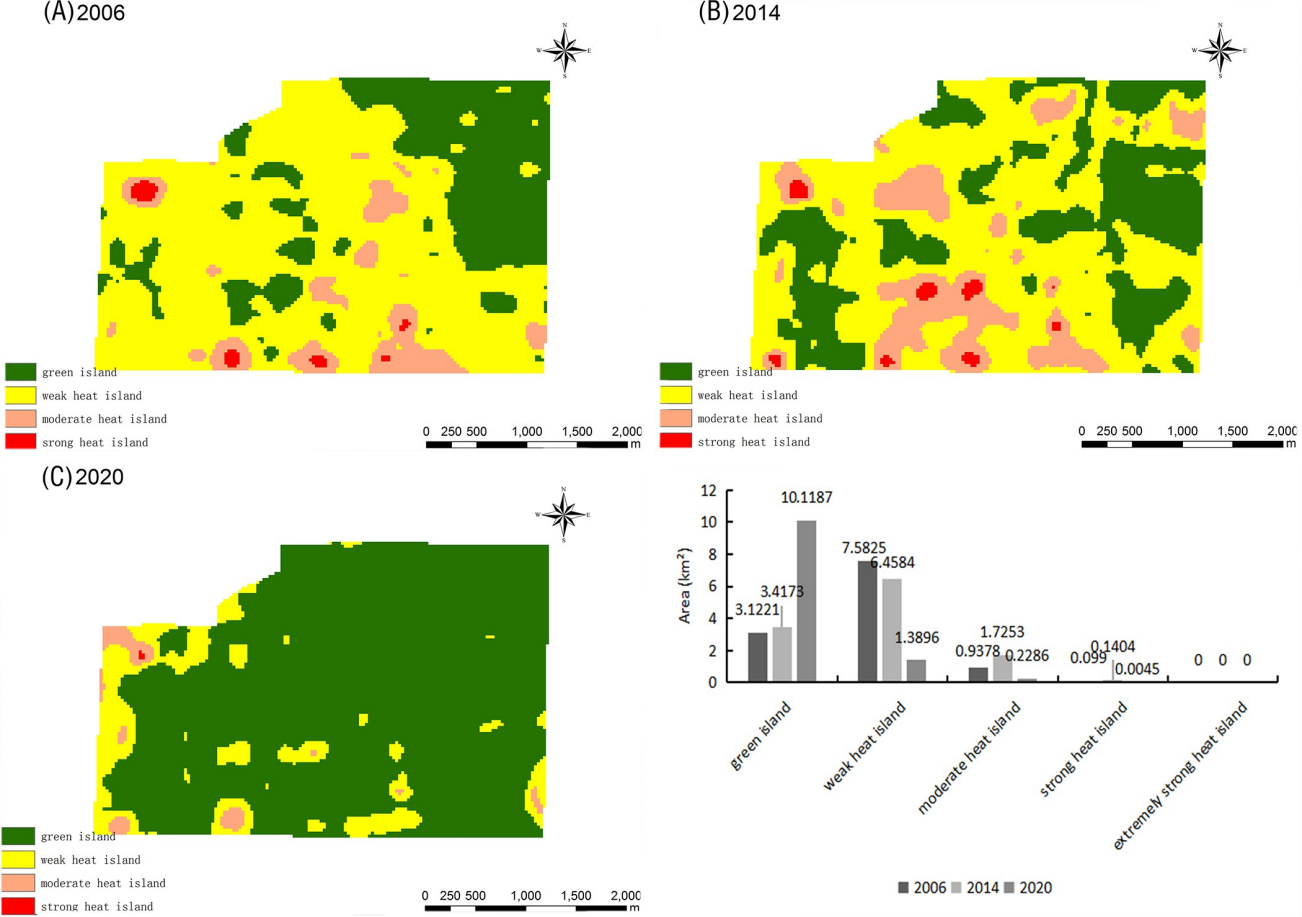
Relative brightness temperature distribution map and area change map of the West University Town in 2006 (A), 2014 (B), and 2020 (C).

The Zhengzhou West University Town is located in the Zhongyuan District. A park was created at this location in 2001 to promote the Zhengzhou National High-Tech Development Zone. As shown in Fig 16, heat island changes were consistent between 2006–2014: weak heat islands dominated, green islands decreased yearly, and moderate heat islands increased yearly. By 2020, the West University Town’s heat island and vegetation coverage had changed dramatically: the green island area was almost equal to the whole area, and the areas of weak, moderate, and strong heat islands had decreased, indicating that urbanization construction in this area had stopped, while the construction and development of urban forest had started. As a result of these measures, the green island area had increased significantly and the ecological environment had improved greatly.

**Fig 17:**
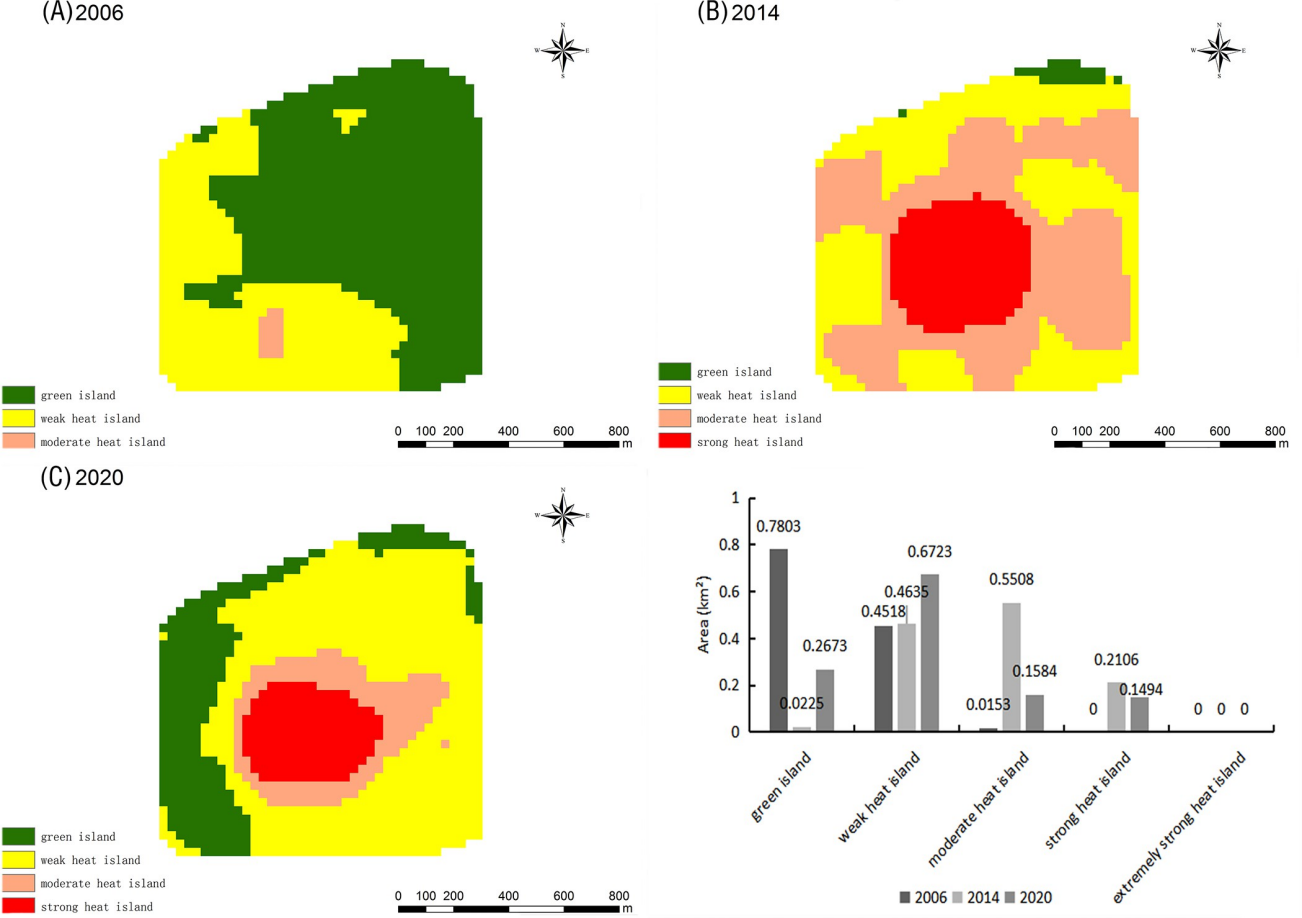
Relative brightness temperature distribution map and area change map of the New Zhengzhou Station Comprehensive Transportation Hub in 2006 (A), 2014 (B), and 2020 (C).

The New Zhengzhou Station Comprehensive Transportation Hub, whose foundation stone was officially laid in 2007, is located in the Guancheng Huizu District. As shown in Fig 17, the TR at this location in 2006 was dominated by green islands. According to the Zhengzhou City Master Plan (2010–2020) (revised in 2017), the Zhengzhou Station Comprehensive Transportation Hub transformed into a key construction area. The largest moderate heat island area was observed in 2014; moreover, a strong heat island of 0.21 km^2^ was observed at that time. This indicates that the ecology of the new Zhengzhou Station integrated transport hub was severely damaged in 2014. By 2020, the green island area had increased, the area of the weak heat islands had decreased, and the area of strong heat islands had also decreased as a result of effective mitigation measures.

**Fig 18:**
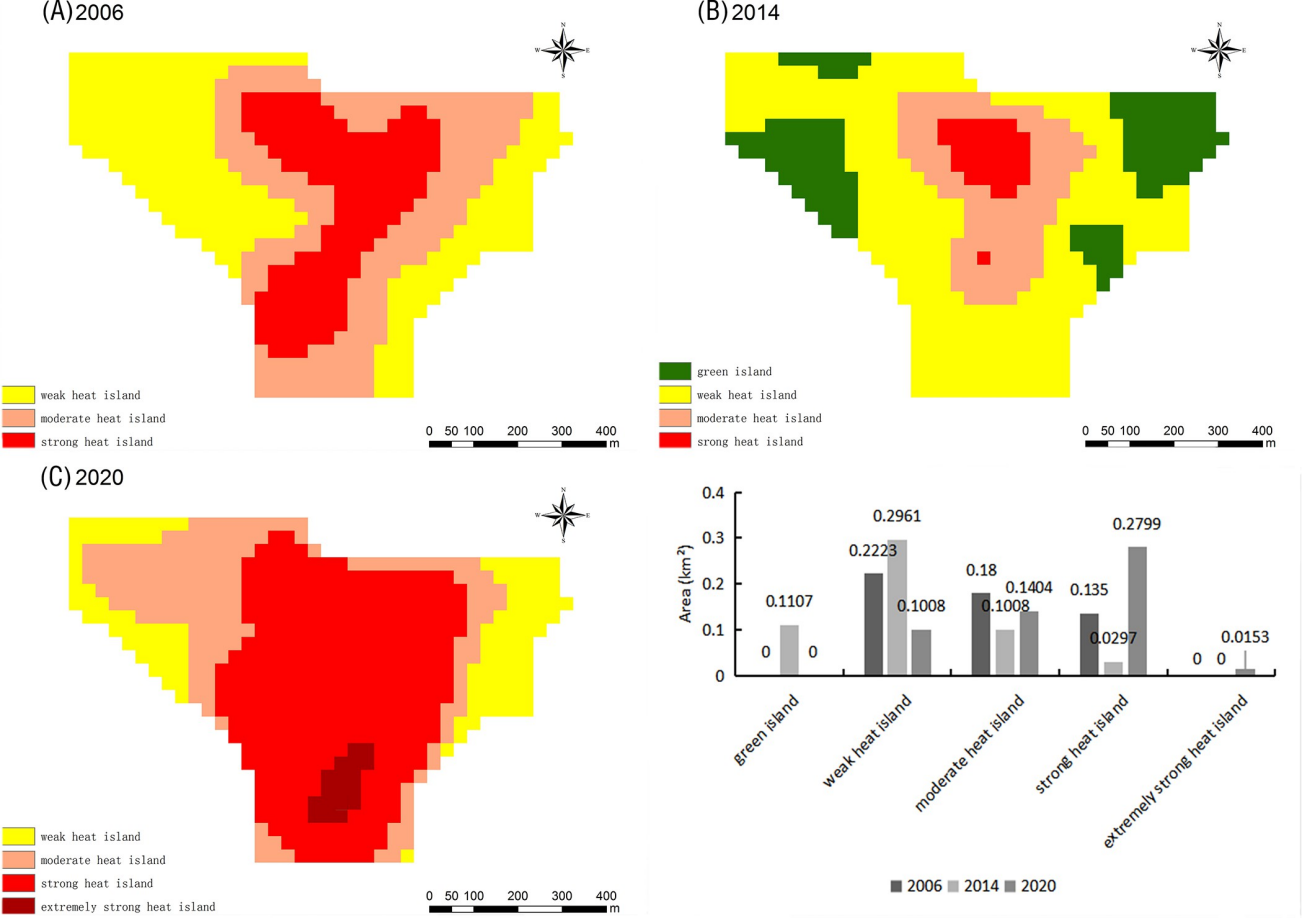
Relative brightness temperature distribution map and area change map of the Zhengzhou Luyuan Kitchen Waste Treatment Company in 2006 (A), 2014 (B), and 2020 (C).

The Zhengzhou Luyuan Kitchen Waste Treatment Company, shown in Fig 18, is located in the Erqi District. Between 2006–2014, this area was dominated by weak heat islands, green islands appeared and increased, and both moderate and strong heat islands decreased, indicating an improvement of the ecological environment over time. However, in 2020, the Luyuan Kitchen Waste Treatment Company coincided with one of the two extremely strong heat island patches of Zhengzhou City, the green island disappeared, and the area of strong heat islands increased, indicating that the ecological environment faced unprecedented challenges at this location in 2020. Therefore, mitigation measures are urgently needed here to improve the thermal environment.

### Limitations

This innovative study on the impact of urban heat island by both urbanization and urban forestry presents limitations. 1) Since we considered Landsat images, whose data accuracy is not high, the results are suitable for macroscopic research (large scale studies) only: the resolution is not sufficient for considerations at the administrative or neighborhood scales. 2) Remote sensing data should be combined with actual surface observation data (e.g., forest structure, vegetation type, and actual surface temperature) to improve the applicability of the research results and the operability of future planning.

## Conclusion

Based on Landsat remote sensing images collected between 2006–2020, we calculated the surface temperature and the vegetation index for the urban area of Zhengzhou City. Geospatial analysis and landscape pattern analysis techniques were integrated to assess the impact of urban construction and urban forestry on the UHI effect in Zhengzhou City, which has been considered a national forest city and the “green core” of the Central Plains Forest City Cluster for the past 15 years. The results showed that, between 2006–2014, Zhengzhou’s UHI deteriorated due to the continuous expansion of urban construction land, changes in land coverage of the urban substrate, landscape fragmentation, and negative changes in vegetation coverage. In this context, strong and very strong heat island patches coincided with industrial agglomerations, indicating the latter had a certain influence on the intensification of the UHI effect. However, between 2014–2020, the UHI effect was mitigated due to reasonable urban planning and construction. In this context, vegetation coverage increased, suggesting that urban forest construction can significantly reduce the UHI effect. Urban forest construction is influenced by vegetation coverage and policy guidance; notably, if urban forest construction preponderates over urbanization construction, the UHI effect will be weakened. We can hence conclude that urbanization construction and urban forestry construction deeply influence the UHI effect. We did not only analyze the spatial and temporal evolutionary characteristics of the urban heat island effect and of vegetation cover in Zhengzhou City, but also quantitatively analyzed the relationship between urbanization, the urban forest, and the urban heat effect. In particular, our results on the two-way effects of urbanization and urban forest on the urban heat island effect in Zhengzhou City during the past 15 years will support the implementation of new solutions for urban heat island mitigation and landscape pattern design. Although we only evaluated construction effectiveness of urbanization and urban forestry, analyzing its causes would provide important references for future urban construction, planning, and decision making.

## Supporting information

S1 Data (ZIP)

## References

[1] Kushwaha S, Nithiyanandam Y. The Study of Heat Island and its Relation With Urbanization in Gurugram, DELHI NCR for the Period of 1990 to 2018. Int Arch Photogramm Remote Sens Spatial Inf Sci. 2019;XLII-5/W3: 49–56. doi: 10.5194/isprs-archives-XLII-5-W3-49-2019

[2] Yao Y, Chen X, Qian J. esearch progress on the thermal environment of the urban surfaces. Acta Ecologica Sinica. 2018;38 (3): 1134–1147. doi: 10.5846/stxb201611022233

[3] Li S, Zhao Z, Wang Y. Urbanization Process and Effects of Natural Resource and Environment in China: Research Trends and Future Directions. 2009;28:63–70. doi: 10.11820/dlkxjz.2009.01.009

[4] Rizwan AM, Dennis LYC, Liu C. A review on the generation, determination and mitigation of Urban Heat Island. Journal of Environmental Sciences. 2008;20: 120–128. doi: 10.1016/s1001-0742(08)60019-4 18572534

[5] Morabito M, Crisci A, Messeri A, Orlandini S, Raschi A, Maracchi G, et al. The impact of built-up surfaces on land surface temperatures in Italian urban areas. Science of the Total Environment. 2016;551–552: 317–326. doi: 10.1016/j.scitotenv.2016.02.029 26878643

[6] Mohajerani A, Bakaric J, Jeffrey-Bailey T. The urban heat island effect, its causes, and mitigation, with reference to the thermal properties of asphalt concrete. Journal of Environmental Management. 2017;197: 522–538. doi: 10.1016/j.jenvman.2017.03.095 28412623

[7] He B-J, Wang J, Liu H, Ulpiani G. Localized synergies between heat waves and urban heat islands: Implications on human thermal comfort and urban heat management. Environmental Research. 2021;193: 110584. doi: 10.1016/j.envres.2020.110584 33285157

[8] He B-J, Wang J, Zhu J, Qi J. Beating the urban heat: Situation, background, impacts and the way forward in China. Renewable and Sustainable Energy Reviews. 2022;161: 112350. doi: 10.1016/j.rser.2022.112350

[9] Zhao H, Tan J, Ren Z, Wang Z. Spatiotemporal Characteristics of Urban Surface Temperature and Its Relationship with Landscape Metrics and Vegetation Cover in Rapid Urbanization Region. Complexity. 2020;2020: 1–12. doi: 10.1155/2020/7892362

[10] Peng S, Zhou K, Ye Y, Su J. Research progress in urban heat island. Ecology and Environment. 2005; 574–579. doi: 10.16258/j.cnki.1674-5906.2005.04.024

[11] Qiu G, Zhang X. China’s Urbanization and Its Ecological Environment Challenges in the 21st Century. Advances in Earth Science. 2019;34: 640–649. doi:CNKI:SUN:DXJZ.0.2019-06-012

[12] Chen A, Yao X, Sun R, Chen L. Effect of urban green patterns on surface urban cool islands and its seasonal variations. Urban Forestry & Urban Greening. 2014;13: 646–654. doi: 10.1016/j.ufug.2014.07.006

[13] Shih W. Greenspace patterns and the mitigation of land surface temperature in Taipei metropolis. Habitat International. 2017;60: 69–80. doi: 10.1016/j.habitatint.2016.12.006

[14] Cui F, Shao F, Qi F, Wang Y, Zhang T, Yu H. Research advances in the influence of vegetation on urban heat island effect. Journal of Zhejiang A&F University. 2020;37: 171–181. doi: 10.11833/j.issn.2095-0756.2020.01.023

[15] Bowler DE, Buyung-Ali L, Knight TM, Pullin AS. Urban greening to cool towns and cities: A systematic review of the empirical evidence. Landscape and Urban Planning. 2010;97: 147–155. doi: 10.1016/j.landurbplan.2010.05.006

[16] Cui Y. Analysis on the Relationship between the Urban Forest Spatial Patterns and Heat Island Effect in Wuhan. Master’s thesis, Huazhong Agricultural University. 2011. Available: https://kns.cnki.net/KCMS/detail/detail.aspx?dbcode=CMFD&dbname=CMFD2012&filename=1011405067.nh&v=

[17] Wang X. On the cool island effect and its impact factors of urban forest: a case study of central district in Changzhou. Master’s thesis, Nanjing Agricultural University. 2018. Available: https://kns.cnki.net/KCMS/detail/detail.aspx?dbcode=CDFD&dbname=CDFDLAST2020&filename=1020037236.nh&v=

[18] Li X, Li T, Qiu K, Jiang S, Jia B. Relationship between Patterns of Urban Forest Patches and Their Cooling Effects———A Case Study of Beijing Urban Area. Scientia Silvae Sinicae. 2021;57: 32–42. doi: 10.11707/j.1001-7488.20210404

[19] Gao M, Jia B, Wang C, Sun Z. Relationship between Urban Forest Canopy Cover and Heat Island Effect in Xiamen Island. Scientia Silvae Sinicae. 2014;50: 63–68. doi: 10.11707/j.1001-7488.20140309

[20] Zhou W, Cao F, Wang G. Effects of Spatial Pattern of Forest Vegetation on Urban Cooling in a Compact Megacity. Forests. 2019;10: 282. doi: 10.3390/f10030282

[21] Jiao M, Zhou W, Qian Y, Wang J, Zheng Z, Hu X. Influences of patch size on the cooling capacity of urban greenspace: progresses, problems and perspectives. Acta Ecologica Sinica. 2021;41. doi: 10.5846/stxb202006191609

[22] Jia B, Qiu K. The cooling effect of plain afforestation in the Beijing Project and its remote sensing-based valuation. Acta Ecologica Sinica. 2017;37. doi: 10.5846/stxb201508221755

[23] Zheng J, Zhang J, Li Y. Heat island effect and human body comfortable degree in Zhengzhou city. Chinese Journal of Applied Ecology. 2005; 1838–1842. http://ir.casnw.net/handle/362004/5281 16422500

[24] Duan J, Song X, Zhang X. Spatiotemporal variation of urban heat island in Zhengzhou City based on RS. Chinese Journal of Applied Ecology. 2011;22: 165–170. doi: 10.13287/j.1001-9332.2011.0003 21548304

[25] Chen Y. Temporal and Spatial Variation of the Imperious Surface Area in Zhengzhou City and its Relationship With Thermal Environment. Master’s thesis, Henan University. 2017. Available:https://kns.cnki.net/KCMS/detail/detail.aspx?dbcode=CMFD&dbname=CMFD201801&filename=1017231250.nh&v=

[26] Min M. Study on Spatial一Temporal Distribution and Causes of Heat Island Effect in Zhengzhou. Master’s thesis, Henan University. 2017. Available: https://kns.cnki.net/KCMS/detail/detail.aspx?dbcode=CMFD&dbname=CMFD201801&filename=1017231178.nh&v=

[27] Zhang F, Bai T, Wang S. Spatial-temporal Evolution Characteristics of the Urban Heat Island Effect in Zhengzhou. Henan Science. 2018;36: 1274–1280. doi: 10.3969/j.issn.1004-3918.2018.08.022

[28] Zhang X. Research on the Heat Island Effect of Zhengzhou City Based on Landsat. Master’s thesis, Northeast Normal University. 2018. Available: https://kns.cnki.net/KCMS/detail/detail.aspx?dbcode=CMFD&dbname=CMFD201901&filename=1018707393.nh&v=

[29] Geng L. Study on the temporal and spatial evolution of heat islandeffect in the central city of Zhengzhou from the perspective of Landscape Architecture. Master’s thesis, Beijing Forestry University. 2020. Available: https://kns.cnki.net/KCMS/detail/detail.aspx?dbcode=CMFD&dbname=CMFD202101&filename=1020338523.nh&v=

[30] Lu Y, Liang Y, Lu S, et al. Spatialization of carbon emissions in Guangzhou City by combining Luojia1-01 nighttime light and urban functional zoning data[J]. Journal of Geo-information Science. 2022;24(6):1176–1188. doi: 10.12082/dqxxkx.2022.210610

[31] Qin Z, Minghua Z, Karnieli A, Berliner P. Mono-window Algorithm for Retrieving Land Surface Temperature from Landsat TM6 data. Acta Ecologica Sinica. 2001;456–466. CNKI:SUN:DLXB.0.2001-04-008

[32] Chibuike EM, Ibukun AO, Abbas A, Kunda JJ. Assessment of green parks cooling effect on Abuja urban microclimate using geospatial techniques. Remote Sensing Applications: Society and Environment. 2018;11: 11–21. doi: 10.1016/j.rsase.2018.04.006

[33] Van De Griend AA, Owe M. On the relationship between thermal emissivity and the normalized difference vegetation index for natural surfaces. International Journal of Remote Sensing. 1993;14: 1119–1131. doi: 10.1080/01431169308904400

[34] Weng Q, Lu D, Schubring J. Estimation of land surface temperature−vegetation abundance relationship for urban heat island studies. Remote Sensing of Environment. 2004;89: 467–483. doi: 10.1016/j.rse.2003.11.005

[35] Li L, Xu S, Wang H, et al. Urban heat island effect based on urban heat island source and sink indices in Shenyang, Northeast China. Chinese Journal of Applied Ecology. 2013;24(12): 3446–3452. doi: 10.13287/j.1001-9332.2013.0578 24697063

[36] Huang C, Li D, Chen Q, Wang X, The effect and mechanism of sponge city construction on heat island mitigation: A case study of Jiaxing. Chinese Journal of Ecology. 2020;39(2): 625–634. doi: 10.13292/j.1000-4890.202002.018

[37] Jia B, Wang C, Qiu K, Cheng J. Dynamic Change of Urban Heat Island Effect in Anshan City. Journal of Northeast Forestry University. 2010;38: 125–127. doi: 10.13759/j.cnki.dlxb.2010.04.002

[38] Lee PS-H, Park J. An Effect of Urban Forest on Urban Thermal Environment in Seoul, South Korea, Based on Landsat Imagery Analysis. Forests. 2020;11: 630. doi: 10.3390/f11060630

[39] Li Y, Jia K, Wei X, Yan Y, Sun J, Mou L. Fractional vegetation cover estimation in northern China and its change analysis. Remote Sensing for Land & Resources. 2015;27: 112–117. doi: 10.6046/gtzyyg.2015.02.18

[40] Feng X, Zhou Z, Li F, Li M. Spatiotemporal differentiation of thermal landscape pattern in Xianyang City driven by the integration of Xi’an and Xianyang. J.Xi’an Univ.Arch. & Tech. (Natural Science Edition). 2021;53: 413–420. doi: 10.15986/j.1006-7930.2021.03.013

[41] Wu J. Landscape Ecology-Concepts and Theories[J]. Chinese Journal of Ecology. 2000(01):42–52. doi: 10.13292/j.1000-4890.2000.0008

[42] Zhao Z. the changes of temperature and the effects of the urbanization in China in the last 39 years. Meteorology.1991;14–17. doi: 10.7519/j.issn.1000-0526.1991.4.003

[43] Zhuo L, Chen J, Shi P, Gu Z, Fan Y, Ichinose T. Modeling population Density of China in 1998 Based on DMSP/OLS Nighttime Light Image. Acta Geographica Sinica. 2005;266–276. doi: 10.3321/j.issn:0375–5444.2005.02.010

[44] Cheng B, Zhou Z, Qian X, Zhang Z, Zhang Y. Urban Heat Island Characteristics and Causes in Zhengzhou. Henan Meteorology. 1997;20–22. doi: 10.16765/j.cnki.1673-7148.1997.01.012

[45] Ge Y, Xin B, Li X. Urban forest construction based on ecosystem service function improvement in warm temperate semi-humid areas. Journal of Beijing Forestry University.2020;42(1):127−141. doi: 10.12171/j.1000–1522.20180433

[46] Yan Z, Zhou D, Zhang L. Contrasting surface thermal environmental effects of urban and agricultural lands in three major urban agglomerations in China. Acta Ecologica Sinica. 2021;41(22):8870一8881. doi: 10.5846/stxb202007251947

[47] Liu Y, Chen Y, Zou C, Li H, Li X. Alleviating Heat Island Effect of Urban Artificial Rivers in Shijiazhuang,Hebei Province. Journal of Ecology and Rural Environment. 2021;37(11):1378一1385. doi: 10.19741/j.issn.1673-4831.2021.0377

[48] Xu Y, Liu Z, Wang J, Hong H. Research on Influencing Mechanism of Regional Difference of Green Rate of Built District of Cities Along Land-Bridge Passage Based on Transect-Geodetector. Landscape Architecture. 2019;26(8):71–76. doi: 10.14085/j.fjyl.2019.08.0071.06

[49] Xu Y, Cheng Y. Research on Spatial Spillover Effect of Urban Green Space Construction in China: Based on the Date of 286 Cities at Prefecture Level or Above. Ecological Economy.2018;34(06):163–167+193.doi:CNKI:SUN:STJJ.0.2018-06-030

[50] Sun Y, Wang H, Li G, Luan Q, Cao Y. Study and influence factors of heat island spatio−temporal changes in Beijing from 2000 to 2019. Environmental Ecology. 2020;2 (8),43~50. Available:https://kns.cnki.net/kcms/detail/detail.aspx?dbcode=CJFD&dbname=CJFDLAST2020&filename=HJSX202008011&uniplatform=NZKPT&v=y3SGqWn53_xo0VQ_XLI1n99Hs9AGLp8ToBI6h-bLP3EK00DEf-3O8EUBNPacXYfL

[51] Zhang M, Ma H, Lin H, Li H, Wang Y. Mitigation effect of different cool roof schemes on thermal environment of urban agglomeration. Climate Change Research. 2021;17 (1): 45–57. doi: 10.12006/j.issn.1673-1719.2020.163

[52] Wang C. Development Scope and Research Scale of Urban Forest in China. Journal of Chinese Urban Forestry. 2021;19: 1–5. doi: 10.12169/zgcsly.2021.08.24.0001

[53] Ding S, Zhang J, Liu X, Dong F. Quantitative Relationship Between Urban Development and Heat Island Effect for Harbin City. Advances in Climate Change Research. 2008;230–234. doi: 10.12006/j.issn.1673-1719.2020.163

[54] Ma M, Xue F, Dang A, Li X, Hu T.Study on the spatial-temporal change of vegetation coverage between the belts of Beijing’s main urban area based on dynamic remote sensing data. Journal of Environmental Engineering Technology. 2019;9: 404–413. doi: 10.12153/j.issn.1674-991X.2019.05.141

[55] Zhang Y, Cao F, Huang L, Zhou Y, Wang J. Spatial Differentiation Pattern of Urban Open Space Vegetation Coverage—A Case Study of Dalian Core Urban Area. Journal of Northwest Forestry University. 2020;35(2):252–259. doi: 10.3969/j.issn.1001-7461.2020.02.38

[56] Chen X, Li L, Wang J. Heat Island Effect Mitigation by Urban Green Space System: A Case Study of Taizhou City. Ecology and Environmental Sciences. 2015;24(4): 643–649. doi: 10.16258/j.cnki.1674-5906.2015.04.015

[57] Wang X, Wei X, Zou H. Research progress about the impact of urban green space spatial pattern on urban heat island. Ecology and Environmental Sciences. 2020;29(9): 1904–1911. doi: 10.16258/j.cnki.1674-5906.2020.09.024

